# Evidence boundaries and integrated disease management of microbial disease risks in protected *Cymbidium faberi* production: a translational framework and validation agenda

**DOI:** 10.3389/fpls.2026.1952084

**Published:** 2026-10-09

**Authors:** Yufeng Deng, Dongfeng Wei, Shichao Gu, Min Yu, Jinzhi Yao, Meicheng Zhao, Lei Qin, Taiji Kou

**Affiliations:** 1 College of Urban Construction, Luoyang Polytechnic, Luoyang, China; 2 Agricultural Resources Research Center, Institute of Genetics and Developmental Biology, Chinese Academy of Sciences, Shijiazhuang, China; 3 College of Agriculture, Henan University of Science and Technology, Luoyang, China

**Keywords:** biological control, *Cymbidium faberi*, disease complex, integrated disease management, orchid diseases, plant microbiome, plant pathogen detection, protected cultivation

## Abstract

*Cymbidium faberi* (*C. faberi*) is an economically and culturally important orchid in East Asia, yet direct species-specific evidence for microbial diseases under protected cultivation remains limited. This critical narrative review synthesizes evidence on orchid diseases, protected-crop disease ecology, plant pathogen detection and diagnosis, biological control, and plant microbiome interactions. Direct *C. faberi* studies comprise one localized anthracnose-oriented report, baseline studies of root- and rhizosphere-associated microorganisms, and candidate antagonistic endophytes. However, they do not establish a contemporary pathogen inventory, species-resolved epidemiology, disease-associated microbiome dynamics, or validated management efficacy. Evidence from related *Cymbidium* species, other orchids, protected crops, and general plant-pathology systems is therefore interpreted according to host proximity, pathogen biology, production environment, and management relevance. Within this synthesis, disease risk is organized provisionally in a disease-complex framework in which environmental filtering, pathogen pressure, host status, and microbiome resilience are considered jointly; the framework does not assume that stable multi-pathogen synergistic pathogenesis has been demonstrated. A node-based translational Integrated Disease Management framework links mother-plant accession, clonal propagation, potting, vegetative growth, flowering, abnormal-batch disposition, and release decisions. The principal research gaps are pathogen isolation and causal confirmation, species-resolved pathogen surveillance, quantitative microclimate and inoculum thresholds, microbiome-function testing, and prospective validation of diagnostic and prediction tools. The framework is intended as a provisional decision-support and research-prioritization structure rather than a validated *C. faberi*-specific management standard.

## 1 Introduction

*Cymbidium faberi* Rolfe (Orchidaceae), commonly known in China as Huilan (hereafter *C. faberi*), and related Chinese *Cymbidium* orchids have ornamental, cultural, and regional economic value in East Asia and contribute to the regional orchid industry (Hinsley et al., 2018; Zhang et al., 2022). As a partial indicator of global trade scale, cut-orchid imports were valued at approximately US$214 million in 2019 (International Trade Centre, 2020); this figure covers the cut-flower segment only and should not be interpreted as the total value of the orchid industry. Wild *C. faberi* is listed as a Class II National Key Protected Wild Plant in China (National Forestry and Grassland Administration and Ministry of Agriculture and Rural Affairs of the People’s Republic of China, 2021). Over the past two decades, the expansion of greenhouse facilities, multi-span production systems, and tissue culture (TC) technology has supported a transition toward more intensive and continuous production (Nongdam et al., 2023; Zhang et al., 2022). These production changes may also favor disease recurrence and facilitate cross-batch transmission through closed canopies, clonal propagation, and repeated batch turnover, potentially constraining plant quality, batch consistency, and production profitability. In this review, “microbial diseases” is used operationally to encompass diseases associated with bacterial, fungal, oomycete, and viral agents. The terms “protected cultivation” and “protected production” refer to commercial cultivation under managed structures such as greenhouses, multi-span greenhouses, and shade houses, rather than to conservation or policy protection.

Asexual propagation may perpetuate *Cymbidium* mosaic virus (CymMV) and Odontoglossum ringspot virus (ORSV) when infected mother plants or clonal material enter the production chain. Latent or asymptomatic infections caused by ORSV and other tobamoviruses may also complicate visual screening and early exclusion (Ilyas et al., 2022; Takahashi et al., 2019). Undetected infections may persist across production stages and affect seedling quality, batch uniformity, and release decisions.

Protected *C. faberi* production presents three interrelated disease-management challenges. First, overlapping symptoms and possible opportunistic or sequential infections complicate etiological diagnosis and pathogen-specific intervention (Dutt et al., 2022; Lamichhane and Venturi, 2015). Second, humid, densely managed environments may prolong infection windows and facilitate within-facility transmission (Daughtrey and Buitenhuis, 2020). Third, repeated reliance on a single mode of action may increase resistance-selection pressure and affect non-target microbial communities (Corkley et al., 2022; Sangiorgio et al., 2022). Together, these factors support an integrated rather than single-pathogen management perspective.

Against this background, this review critically synthesizes evidence on pathogen ecology, disease syndromes, interaction mechanisms, diagnosis, and management relevant to protected *C. faberi* production. Because direct evidence is limited, studies from other *Cymbidium* species, other Orchidaceae, protected ornamental crops, and general plant-pathology systems are interpreted according to host proximity, environmental comparability, pathogen biology, and asexual-propagation risk. The objectives are to define the evidence boundaries of a disease-complex interpretation, develop a node-based translational Integrated Disease Management (IDM) framework, and identify the validation steps required for a calibrated *C. faberi*-specific system. Quantitative data on production scale, protected-cultivation area, and disease-related losses remain scarce; therefore, the production-risk framing relies mainly on production-system logic and the broader orchid and ornamental-crop literature rather than dedicated economic surveys.

## 2 Literature scope and translational interpretation

This article presents a critical narrative review of microbial disease risks and management options relevant to protected *Cymbidium faberi* production. The evidence base was assembled through an iterative, question-driven literature-identification process conducted alongside development of the main sections of the review. Searches of Web of Science, PubMed, Scopus, and the China National Knowledge Infrastructure (CNKI) were used to identify publications relevant to specific topics, including orchid pathogens, molecular and serological diagnostics, greenhouse disease ecology, irrigation and substrate management, biological control, plant-associated microbiomes, and integrated disease management. Relevant publications were also followed through reference tracing and targeted topic-specific searches. The literature considered primarily covered publications from 2000 to June 2026, while earlier studies were retained when they provided relevant pathogen records, diagnostic methods, or foundational biological evidence.

The purpose of this literature-identification process was to identify studies informative for the biological, diagnostic, ecological, and management questions addressed in this review, rather than to construct an exhaustive or formally screened evidence corpus. The searches were therefore not conducted as a single predefined systematic-review query that generated a deduplicated set of candidate records, and no formal title/abstract screening or predefined inclusion/exclusion workflow was used. Because these counts were not recorded during the original literature-identification process, numbers of initially retrieved, screened, and excluded records are not reported or reconstructed retrospectively. The scientific evidence synthesis comprises 82 publications characterized in **Supplementary Table S2**: 81 peer-reviewed journal articles and one book chapter. Because direct pathological evidence for *C. faberi* was scarce, studies involving other *Cymbidium* species, other Orchidaceae, non-orchid greenhouse crops, and general plant-pathology systems were also considered when their host characteristics, pathogen biology, transmission routes, protected-production conditions, or management objectives were relevant to *C. faberi*.

The 82 scientific publications included in the evidence synthesis were subsequently classified descriptively as direct *C. faberi* studies (CF), other *Cymbidium* studies (CY), other Orchidaceae studies (OR), non-orchid greenhouse or nursery studies (GH), or model and general plant-pathology studies (MP). These labels indicate source context and approximate extrapolation distance rather than evidence quality, risk of bias, or formal evidence grade. **Supplementary Table S2** provides a descriptive summary of these 82 scientific publications, including study host, topic, study type, pathogenicity testing, and production-relevant evaluation, to make the source context and limits of extrapolation transparent.

The translational IDM framework was developed by integrating recurrent disease risks, diagnostic requirements, and management responsibilities across seven protected-production nodes. Framework components were incorporated when their biological rationale or operational function was supported either by direct *C. faberi* information or by biologically and operationally relevant studies from related orchids and protected-crop systems. The validation agenda was organized according to the major unresolved issues constraining pathogen attribution, threshold calibration, intervention selection, and production-stage decision-making.

### 2.1 Evidence proximity and limits of inference

Direct *C. faberi*-specific evidence is uneven rather than absent. A localized study from Luanchuan County isolated and identified an anthracnose-associated fungus and screened nine plant extracts *in vitro* (Liu et al., 2009), but its restricted sampling and historical taxonomic resolution do not provide a contemporary pathogen inventory or validated production recommendation. Culture-dependent studies characterized culturable root-endophytic bacteria and recovered diverse root-associated fungi, including *Tulasnella*, *Trichoderma*, *Fusarium*, and *Umbelopsis* (Wang et al., 2015; Yu et al., 2015). Luo et al. (2019) documented geographic differentiation in culturable root-endophytic fungal assemblages, while sequencing-based studies described rhizosphere bacterial and fungal communities and their environmental associations in introduced, domesticated, or wild *C. faberi* populations (Lv et al., 2023a; Du et al., 2025; Wen et al., 2025). Jiang et al. (2023) obtained candidate antagonistic endophytic fungi from *C. faberi* roots, although the subsequent *in vivo* test used *C. goeringii* rather than *C. faberi*.

Collectively, these studies provide direct-host baseline information and one localized disease-oriented report, but remain insufficient to define the contemporary pathogen spectrum, species-level causal relationships across production regions, disease epidemiology, disease-associated microbiome dynamics, or management efficacy under protected production. Most candidate-pathogen, mechanistic, diagnostic, environmental-risk, and management interpretations therefore still depend on evidence from related *Cymbidium* species, other orchids, protected crops, and general plant-pathology systems. The following syndrome synthesis, conceptual model, diagnostic pathway, and node-based IDM framework should be interpreted as biologically informed and testable translational structures rather than calibrated *C. faberi*-specific protocols.

## 3 Disease ecology in *Cymbidium faberi* production systems

In *C. faberi* protected cultivation (greenhouses, multi-span greenhouses, or shade houses), environmental filtering is likely to act as a major modifier of infection risk and disease amplification distinguishing protected cultivation from traditional open-field cultivation, based on evidence from other greenhouse crops and cross-crop climate models. The clasping leaf sheaths, fleshy root system, and high-density potting of *C. faberi* may promote localized high-humidity microenvironments within greenhouses; when canopy ventilation is poor, water films readily persist on leaf surfaces for extended periods. Drawing on the general principles derived from other greenhouse crops and cross-crop climate models, high humidity is associated with increased fungal-disease risk (Mahmoud et al., 2023; Romero et al., 2022). Direct reports in *C. faberi* on this topic remain extremely scarce, and evidence mostly stems from other *Cymbidium* species and protected ornamental crops: greenhouse crops generally indicate that rhizosphere and stem-base rots are more strongly regulated by substrate moisture conditions, and that chronically over-wet or compacted substrates reduce root-zone oxygen content and weaken root vigor, creating infection opportunities for oomycetes, soil-borne fungi, and opportunistic bacteria (Redekar et al., 2020; Bailey-Serres and Voesenek, 2008). Quantitative greenhouse microclimate-substrate-root thresholds remain poorly studied in *C. faberi* and related orchids; leading locally grown cultivars and representative greenhouse types should be combined to establish *C. faberi*-specific temperature-humidity-disease risk thresholds.

The high-frequency clonal division, repotting, and TC propagation of *C. faberi* constitute a major production-mediated transmission pathway within the protected-cultivation pipeline. Viruses such as CymMV and ORSV can accumulate over successive generations via infected mother plants through asexual propagation and spread to new batches; ORSV and other tobamoviruses can additionally form latent or asymptomatic infections, further increasing the difficulty of detection and blockade within greenhouses (Ilyas et al., 2022). A regional survey of symptomatic Chinese *Cymbidium* production samples in Guangdong detected both CymMV and ORSV, including mixed infections, although the results were not resolved specifically for *C. faberi* (Ren et al., 2020). In *C. faberi*, direct field epidemiological studies targeting this transmission chain remain lacking; reports from other *Cymbidium* species and commercial orchids (e.g., *Phalaenopsis*) consistently indicate that mechanical wounds caused by division and repotting provide entry routes for bacteria and fungi, whereas recirculated irrigation water, shared tools, and cross-bench seedling movement may facilitate pathogen transmission throughout an entire production unit (Redekar et al., 2020; Daughtrey and Buitenhuis, 2020). *C. faberi* and related Chinese *Cymbidium* orchids, which rely predominantly on division and TC, would benefit from strengthened mother-plant virus monitoring and propagation-material traceability within protected batch turnover, thereby blocking a transmission chain that may otherwise be amplified under intensive production.

The above protected-cultivation environment and clonal-transmission conditions together indicate that a single-pathogen perspective may be insufficient for interpreting disease risks at the protected-production-system level in *C. faberi*. Consistent with general plant-microbiome principles (Trivedi et al., 2020; Leveau, 2024), plant-associated microbiomes can influence disease progression through colonization resistance, resource competition, and immune induction, whereas greenhouse management measures such as fungicide application, substrate replacement, or drastic temperature-humidity changes may, while suppressing target pathogens, also disturb phyllosphere and rhizosphere community structure, weakening disease-suppressive potential and facilitating secondary colonization by opportunistic pathogens (Arnault et al., 2023; Lv et al., 2023b; Spooren et al., 2024; Emmenegger et al., 2023; Sangiorgio et al., 2022). Direct-host studies have begun to characterize the baseline root and rhizosphere microbiota of *C. faberi*. Culture-based work identified growth-environment-associated differences in culturable root-endophytic bacterial assemblages (Wang et al., 2015), recovered diverse root-associated fungi from *C. faberi* roots (Yu et al., 2015), and documented geographic differentiation in culturable root-endophytic fungal communities across Qin-Ba Mountain populations (Luo et al., 2019). Sequencing studies subsequently described rhizosphere bacterial and fungal communities and their associations with soil physicochemical conditions (Lv et al., 2023a; Du et al., 2025; Wen et al., 2025). These studies were not designed around diseased versus healthy plants, longitudinal disease onset, pathogen-load measurements, or protected-production interventions. Therefore, systematic evidence linking phyllosphere or rhizosphere community dynamics to disease emergence, pathogen pressure, or management response under protected *C. faberi* production remains lacking. Evidence from other orchids and protected crops consequently remains necessary for interpreting microbiome resilience and disease-suppressive function within the proposed disease-complex framework (Leveau, 2024; Dastogeer et al., 2022; Lv et al., 2023b; Spooren et al., 2024). Protected-cultivation microbiome resilience and disease-suppressive function are therefore priority targets for *C. faberi*-specific multi-omics tracking and intervention validation. Accordingly, the disease-complex concept is used here as an organizing framework for symptom overlap, sequential infection, and environment-mediated disease risk, rather than as evidence that a stable multi-pathogen synergistic pathosystem has been demonstrated in *C. faberi*.

## 4 Major disease syndromes and candidate pathogens: a complex perspective

In protected orchid-production systems, some disease syndromes cannot be adequately interpreted through a simple one-pathogen-one-host framework and may present disease-syndrome or complex characteristics (Bag et al., 2024; Dutt et al., 2022). The distinctive plant architecture of *C. faberi*—leaf-sheath clasping and fleshy roots—together with frequent clonal division, repotting, dense pot placement, and protected-house microclimate, may allow pathogens of different taxonomic groups to converge phenotypically under environmental stress. High humidity and water films, over-wet substrates, poor ventilation, high temperature, prolonged leaf wetness, crowded canopies, senescent flowers, and transport-related stress may open ecological niches for opportunistic or sequential infection. However, direct validation of these temporal sequences and interaction mechanisms in *C. faberi* remains limited. Accordingly, this section is organized around disease syndromes, with major syndromes, candidate pathogens, diagnostic methods, study contexts, and major evidence limitations summarized in **Table 1**. The likely organ- and stage-specific high-risk windows are visualized in the spatiotemporal framework (**Figure 1**).

**Table 1:** Overview of major disease syndromes and candidate pathogens in *C. faberi* protected production.

Disease syndrome	Candidate pathogens	Main symptoms	Common diagnostic methods	Relevant references	Evidence limitations	Study context	Reported country/region of empirical evidence
Soft-rot/wet-rot complex	*Pectobacterium*, *Dickeya*, *Pythium*, *Phytophthora*	Water-soaked rot of pseudobulbs/new shoots, root rot, foul odor	Isolation and culture, PCR/qPCR, and validated target-specific molecular assays	(Charkowski, 2018; Zhou et al., 2021; Bag et al., 2024)	Limited direct pathogenicity validation and pathogen-interaction temporal data in *C. faberi*	OR+MP	Canada
Leaf spot and anthracnose	*Colletotrichum* spp.; *Fusarium proliferatum*; *Pestalotiopsis* sp.; *F. oxysporum*; bacterial leaf spot or rot, including *Burkholderia gladioli*; *Cercospora* and *Alternaria* as broader ornamental-crop differentials	Leaf lesions, leaf-tip necrosis, latent infection	Isolation and culture, morphology, multilocus sequencing, PCR or multiplex PCR, and pathogenicity testing	(Liu et al., 2009; Park et al., 2020; Huang et al., 2022; Kim et al., 2024; Moon et al., 2017; Guarnaccia et al., 2021)	One localized *C. faberi* anthracnose-oriented study is available, but its taxonomic resolution and production validation are limited; multi-region, cultivar-resolved causal and epidemiological studies remain lacking.	CF+CY+GH	China; Korea; Taiwan
Floral disease/gray mold	*Botrytis cinerea*	Flower rot, gray mold, petal rot	Microscopy, PCR	(Muñoz et al., 2019; Bika et al., 2021)	Limited data on floral microecology and epidemic conditions in *C. faberi*	GH	Colombia
Viral diseases	CymMV (*Potexvirus*), ORSV (*Tobamovirus*)	Mosaic, ringspot, deformation, asymptomatic	Serological and targeted nucleic-acid assays; RT-RPA for rapid screening; HTS for unresolved cases	(Ren et al., 2020; Eun et al., 2000; Sun et al., 2024; Villamor et al., 2019)	A regional Chinese *Cymbidium* survey supports the occurrence of single and mixed CymMV/ORSV infection, but symptom-based sampling and unresolved host-species composition preclude *C. faberi*-specific prevalence inference; synergistic pathogenic effects in *C. faberi* remain unvalidated.	CY+OR+MP	China
Root/stem-base/pseudobulb rot complex	*Pythium*, *Phytophthora*, *Fusarium oxysporum*, *Mucor hiemalis*	Root browning, root rot, pseudobulb water-soaking, plant wilt	Isolation and culture, qPCR, sequencing, pathogenicity testing	(Sen et al., 2006; Huang et al., 2020; Chang et al., 2026; Bag et al., 2024; Redekar et al., 2020)	Rhizosphere pathogen dynamics and co-infection mechanisms in *C. faberi* await validation	CY+OR+GH	India; Taiwan; United States

Study context: CF, direct *Cymbidium faberi* studies; CY, other *Cymbidium* species; OR, other Orchidaceae; GH, non-orchid greenhouse or nursery crops; MP, model plants and other general plant-pathology systems. These abbreviations identify the original host or production context and do not represent formal evidence grades. Candidate pathogens listed here should be interpreted as priorities for differential diagnosis, pathogen isolation, and pathogenicity testing rather than as confirmed pathogens of *C. faberi*. Disease-syndrome categories are not mutually exclusive; overlapping candidate pathogens may produce different symptom profiles depending on the infected organ, production stage, environmental conditions, and infection sequence. Reported country/region refers to the location of empirical study material or production sampling among the cited sources; review-only sources are not assigned a country.

**Figure 1:**
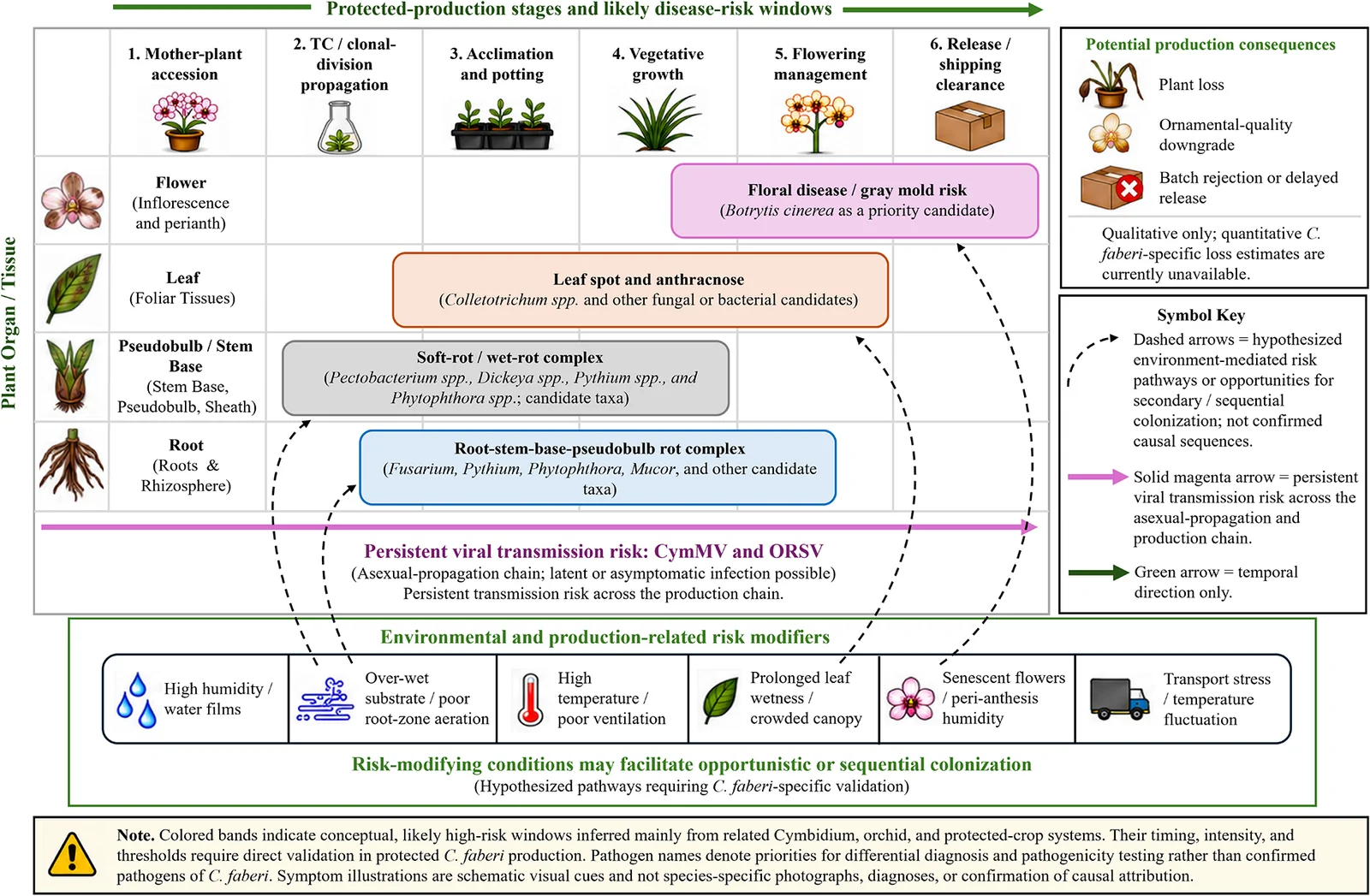
Conceptual spatiotemporal overview of major microbial disease syndromes, likely disease-risk windows, and potential production consequences in protected *Cymbidium faberi* production. Colored bands indicate conceptual, likely high-risk windows across production stages and plant organs. Schematic symptom illustrations provide visual cues for affected flowers, leaves, pseudobulb/stem bases, and roots but are not species-specific photographs, diagnoses, or confirmation of causal attribution. The magenta horizontal arrow indicates persistent transmission risk of CymMV and ORSV across the asexual-propagation and production chain, and the green arrow indicates temporal direction only. Black dashed arrows indicate hypothesized environment-mediated risk pathways or opportunities for secondary or sequential colonization and do not represent confirmed causal sequences. Pathogen names denote priorities for differential diagnosis and pathogenicity testing rather than confirmed pathogens of *C. faberi*. Potential production consequences—plant loss, ornamental-quality downgrade, and batch rejection or delayed release—are qualitative because quantitative *C. faberi*-specific disease-loss estimates are currently unavailable. The timing, intensity, and thresholds of the illustrated risk windows require direct validation under protected *C. faberi* production.

### 4.1 Soft-rot complex: pseudobulb and new-shoot rot

Soft-rot diseases are characterized by the water-soaked disintegration of pseudobulbs and tender new shoots, with rapid disease progression and marked tissue destruction, making soft rot a potentially destructive syndrome in protected orchid production. In *C. faberi*, systematic etiological and epidemiological studies directly addressing pseudobulb soft rot are currently lacking; however, reports from related *Cymbidium* species and commercial orchids provide clues for pathogen candidates: bacterial soft rot has been reported in association with members of the Pectobacteriaceae (*Pectobacterium* and *Dickeya*); Zhou et al. (2021) first reported *Dickeya fangzhongdai* causing soft rot in an orchid in Canada; this taxon should be included among the differential taxa where isolation, molecular detection, or epidemiological evidence supports its consideration (Charkowski, 2018; Zhou et al., 2021). Wet rot or black rot is commonly associated with *Pythium* spp. and *Phytophthora* spp.; its occurrence is closely related to chronic waterlogging of the protected-cultivation substrate and rhizosphere hypoxia, and zoospores can spread within nurseries via water films and recirculated irrigation water (Bag et al., 2024; Redekar et al., 2020). Current evidence cannot yet determine whether bacteria and oomycetes form a stable temporal sequence of secondary infection in *C. faberi*; this causal chain still requires validation through sequential sampling and co-inoculation experiments (Dutt et al., 2022; Lamichhane and Venturi, 2015).

### 4.2 Leaf spot and anthracnose: leaf-lesion formation and latent infection

Foliar diseases in *C. faberi* should be treated as a differential-diagnosis syndrome involving both fungal and bacterial candidates. An early localized study from Luanchuan County investigated anthracnose in *C. faberi*, reported isolation and identification of an anthracnose-associated fungus, and screened nine plant extracts for *in vitro* inhibition (Liu et al., 2009). However, because it was geographically localized and predates current multilocus fungal-taxonomy standards, the reported taxonomic assignment and candidate plant extracts require re-evaluation through modern identification, standardized pathogenicity testing, phytotoxicity assessment, and protected-production trials.

Jiang et al. (2023) subsequently isolated endophytic fungi from healthy roots of *C. faberi* ‘Hongxiangfei’ and identified strains with *in vitro* inhibitory activity against *Colletotrichum gloeosporioides* and *F. oxysporum*. The subsequent *in vivo* suppression test was conducted on *C. goeringii* ‘Green Cloud’, not on *C. faberi*. This study therefore provides *C. faberi*-derived candidate antagonists and cross-species proof of concept, but it does not independently establish *Colletotrichum gloeosporioides* or *F. oxysporum* as current causal pathogens of *C. faberi*.

Studies of other *Cymbidium* species provide stronger causal and stage-specific evidence. Park et al. (2020) confirmed that *Colletotrichum cymbidiicola* can cause anthracnose on *Cymbidium* orchids. Huang et al. (2022) confirmed *Fusarium proliferatum* as the causal agent of fleck spot on Oriental *Cymbidium*, based on morphology, ITS and TEF-1α sequence data, artificial inoculation, and re-isolation. In that study, young shoots of *C. ensifolium*, *C. sinense*, and *C. goeringii* developed brown-to-black fleck lesions, whereas mature leaves remained symptomless after inoculation. These findings support inclusion of *F. proliferatum* in differential diagnosis and provide a biological basis for a young-shoot risk window in related *Cymbidium* species, but they should not be interpreted as a calibrated *C. faberi* disease calendar.

Kim et al. (2024) developed a multiplex PCR assay capable of simultaneously testing *C. kanran* leaf samples for *Colletotrichum gloeosporioides*, *Pestalotiopsis* sp., *F. oxysporum*, and *F. proliferatum*, providing a methodological basis for differential and multi-target screening. Moon et al. (2017) isolated *Burkholderia gladioli* from Korean *Cymbidium* leaves and confirmed pathogenicity, indicating that differential diagnosis of foliar lesions should also include bacterial leaf spot or rot. Fungal genera such as *Cercospora* and *Alternaria* have been associated with opportunistic foliar lesions in broader ornamental-crop systems and should be considered where supported by isolation and pathogenicity testing (Guarnaccia et al., 2021).

Beyond the localized report by Liu et al. (2009), systematic multi-region, multilocus, and cultivar-resolved studies of the *C. faberi* foliar pathogen spectrum remain lacking. Modern re-identification of anthracnose-associated isolates, standardized pathogenicity testing, cultivar-susceptibility comparisons, and evaluation of predisposing protected-production conditions—particularly high-humidity and young-shoot windows—should therefore receive priority.

### 4.3 Floral diseases: high humidity at flowering and gray mold risk

The architecture of *C. faberi* racemes may promote localized humidity at petal-contact surfaces under poorly ventilated protected conditions, providing conditions for the germination and colonization of necrotrophic fungi such as *Botrytis cinerea*. In *C. faberi*, direct reports on floral diseases are currently lacking; evidence from other orchids and greenhouse ornamental crops indicates that high humidity at flowering and the retention of senescent flowers are key predisposing factors for gray mold epidemics (Muñoz et al., 2019; Bika et al., 2021). Floral-disease microecology and epidemic conditions remain comparatively underexplored in orchids; *C. faberi* and related orchids for which the flower is a high-value commercial organ warrant particular attention to flowering-stage greenhouse management, senescent-flower removal, and peri-anthesis dehumidification.

### 4.4 Root-stem-base-pseudobulb rot complex: velamen structure and dysbiosis

In related orchid systems, syndromes involving root rot, stem-base rot, and pseudobulb rot may result from single or combined infections by multiple soil-borne pathogens. No direct systematic study of this syndrome complex has been conducted in *C. faberi*; however, reports from other *Cymbidium* species provide important clues: Sen et al. (2006) reported that *Cymbidium* pseudobulb soft rot can be jointly caused by *Erwinia carotovora* (now mostly reclassified as *Pectobacterium carotovorum*), *Fusarium oxysporum*, and *Mucor hiemalis*; Huang et al. (2020) further confirmed that *F. oxysporum* alone can cause pseudobulb rot in *Cymbidium*. Recent Taiwanese work documented diseases caused by the *Fusarium oxysporum* species complex across cultivated orchid hosts, providing additional *Cymbidium*-relevant evidence for differential diagnosis in *C. faberi* (Chang et al., 2026). Beyond bacteria and *Fusarium*, population studies of oomycetes and other soil-borne fungi in orchid crops also suggest their possible involvement in this complex (Bag et al., 2024; Redekar et al., 2020), although the temporal sequence and interaction mechanisms among these groups in *C. faberi* still require validation. *C. faberi*-specific pathogen monitoring and co-infection validation at the substrate-root level are the corresponding priorities.

### 4.5 Viral diseases and co-infection: the hidden risk of asexual propagation chains

In the *C. faberi* protected-cultivation industry, where clonal division and tissue culture are major propagation methods, CymMV and ORSV may be repeatedly transmitted through infected propagation material and represent an important but easily overlooked production-chain risk. Regionally relevant evidence is available from a survey of symptomatic Chinese *Cymbidium* in Guangdong Province. Among 53 symptomatic leaf samples collected from three production areas, 44 tested positive for CymMV and/or ORSV by DAS-ELISA, and RT-PCR testing of 35 selected samples also detected single and mixed infections (Ren et al., 2020). Because the sampling was symptom-based and the results were not resolved specifically for *C. faberi*, these findings demonstrate the occurrence of CymMV and ORSV in Chinese *Cymbidium* production but should not be interpreted as *C. faberi* prevalence or cultivar-specific epidemiological estimates. Direct *C. faberi* field surveys and cultivar-background data for these viruses remain lacking. Studies of other *Cymbidium* species and commercial orchids nevertheless show that CymMV and ORSV can be detected singly or together within the same plant (Eun et al., 2000; Ren et al., 2020; Sun et al., 2024), while latent or asymptomatic infection can further complicate visual screening and early transmission blockade (Ilyas et al., 2022). Whether the two viruses produce stable synergistic pathogenic effects in *C. faberi*, and whether such effects vary with cultivar background or physiological stage, remain unvalidated. *C. faberi* and related Chinese *Cymbidium* orchids therefore require species-resolved virus surveillance, clean-stock programs, and evaluation of single and mixed infection within mother-plant and clonal-propagation systems.

## 5 Cross-scale interaction mechanisms: pathogen infection strategies, host immune landscape, and microecological regulation

Drawing on general principles of plant-microbiome-pathogen tripartite interactions (Trivedi et al., 2020; Leveau, 2024), the occurrence of *C. faberi* microbial diseases can also be viewed as a process jointly driven by pathogen infection strategies, host immune responses, and microbiome changes acting across different scales. At the cellular and organismal levels, pathogens must breach structural barriers such as the cuticle and velamen and evade or suppress immune responses (Lu et al., 2022; Dodds et al., 2024); at the population level, changes in the composition of rhizosphere and phyllosphere microbial communities influence host disease resistance. Understanding these interactions helps explain the commonly observed “same symptom, different cause” and co-infections in production, and also provides a basis for formulating IDM strategies.

### 5.1 Pathogen infection strategies: enzymatic invasion and immune suppression

Assuming conservation of broad infection principles across orchids, candidate pathogens of *C. faberi* would need to breach physical barriers such as the cuticle, velamen, and leaf sheath and subsequently acquire host nutrients. Direct mechanistic evidence on infection strategies and species-resolved pathogenicity of contemporary *C. faberi* isolates remains limited. Referring to the general pathogenicity models of cross-crop soft-rot diseases and *Colletotrichum* effectors, the pathogenic strategies of different ecological functional groups can be summarized as follows: necrotrophic bacteria (e.g., *Pectobacterium*, *Dickeya*) can produce plant cell-wall-degrading enzymes (PCWDEs) that contribute to tissue maceration and rapid pseudobulb disintegration (Charkowski, 2018); hemibiotrophic *Colletotrichum* spp. rely more on effectors to modulate host immunity and promote colonization (Lu et al., 2022), whereas host recognition of and immune-signaling responses to such effectors follow general principles of plant immunity (Dodds et al., 2024). These mechanisms have been comparatively well studied in model plants and major crops, but direct evidence in *C. faberi* and related *Cymbidium* strains is still lacking; the specific targets and expression dynamics of enzymatic hydrolysis and effector regulation should be validated through *C. faberi* tissue histochemistry, transcriptomics, and pathogenicity assays.

### 5.2 Orchid immune-genomic features and defense trade-offs

Direct studies of the *C. faberi* immune genome are currently lacking; therefore, its immune background can only be extrapolated from comparative genomic evidence in other orchids. Whole-genome analyses of some orchids reveal that gene families encoding nucleotide-binding leucine-rich repeat (NLR) resistance proteins are markedly fewer in number than in several crop lineages (Xue et al., 2020), although whether this pattern equally applies to *C. faberi* awaits validation from *C. faberi* genomic and functional data. Accordingly, persistent viral infection may plausibly alter host physiological or immune status; however, whether this affects subsequent bacterial or fungal infection in *C. faberi* remains untested. Current orchid-relevant evidence more directly supports the occurrence and management significance of latent virus infection than a defined cross-pathogen immune mechanism (Ilyas et al., 2022). The genetic basis of immunity and defense-metabolism trade-offs remains poorly understood in orchids; *C. faberi* would particularly benefit from a high-quality genome and resistance-gene (NLR) mapping to lay the foundation for marker-assisted selection (MAS) and disease-resistance breeding.

### 5.3 Microecological regulation: orchid mycorrhizal interactions and community resilience

Orchid mycorrhizal fungi (OMF), such as *Tulasnella*, can form symbiotic relationships with the roots of many orchids, including other *Cymbidium* species (Yang et al., 2021). Direct *C. faberi* studies provide a baseline for root-associated microbial diversity but not for disease-suppressive function. Wang et al. (2015) documented growth-environment-associated differences in culturable root-endophytic bacteria. Yu et al. (2015) recovered *Tulasnella* together with Umbelopsis, Trichoderma, and Fusarium from *C. faberi* roots, but the limited, isolation-based study did not establish stable mycorrhizal colonization or functional symbiosis. Luo et al. (2019) documented marked geographic differentiation in culturable root-endophytic fungal assemblages across three Qin-Ba Mountain populations. Lv et al. (2023a) and Du et al. (2025) characterized rhizosphere microbial assemblages and their environmental associations, whereas Wen et al. (2025) profiled rhizosphere fungi and isolated root endophytes from wild *Cymbidium* populations. Jiang et al. (2023) identified candidate antagonistic endophytic fungi from *C. faberi* roots, but the subsequent *in vivo* test used *C. goeringii*. Thus, direct-host studies establish that *C. faberi* supports diverse and environmentally responsive root-associated microbial communities and that candidate mycorrhizal or beneficial taxa can be recovered from its roots. However, they do not demonstrate stable OMF-mediated disease suppression or microbiome-based control under protected production. Evidence from other orchid systems indicates that OMF can prime induced systemic resistance (ISR), suggesting a biologically plausible route for disease suppression (Pujasatria et al., 2024). However, substrate replacement, agrochemical use, high temperature and humidity, and repeated vegetative division during clonal propagation may reshape root-associated communities and affect OMF establishment or persistence (Trivedi et al., 2020; Emmenegger et al., 2023). OMF-mediated disease suppression should therefore remain a testable management hypothesis rather than a current production recommendation, pending *C. faberi*-specific taxonomic confirmation, in planta colonization tracking, disease-challenge experiments, and multi-location, multi-season protected-production validation.

### 5.4 A translational conceptual model of *Cymbidium faberi*-pathogen-microbiome interactions

**Figure 2** integrates environmental filtering, pathogen pressure, host status, microbiome resilience, and management interventions within a hypothesis-organizing framework. Candidate indicators include greenhouse relative humidity, leaf-wetness duration, substrate moisture, ventilation, pathogen load, host physiological status, microbial-community attributes, and management records. These indicators combine operational monitoring variables with research-oriented validation metrics and are not assumed to have equivalent readiness for routine production. Their weights, critical values, pathway strengths, and pathogen-specific thresholds require direct calibration under protected *C. faberi* conditions.

**Figure 2:**
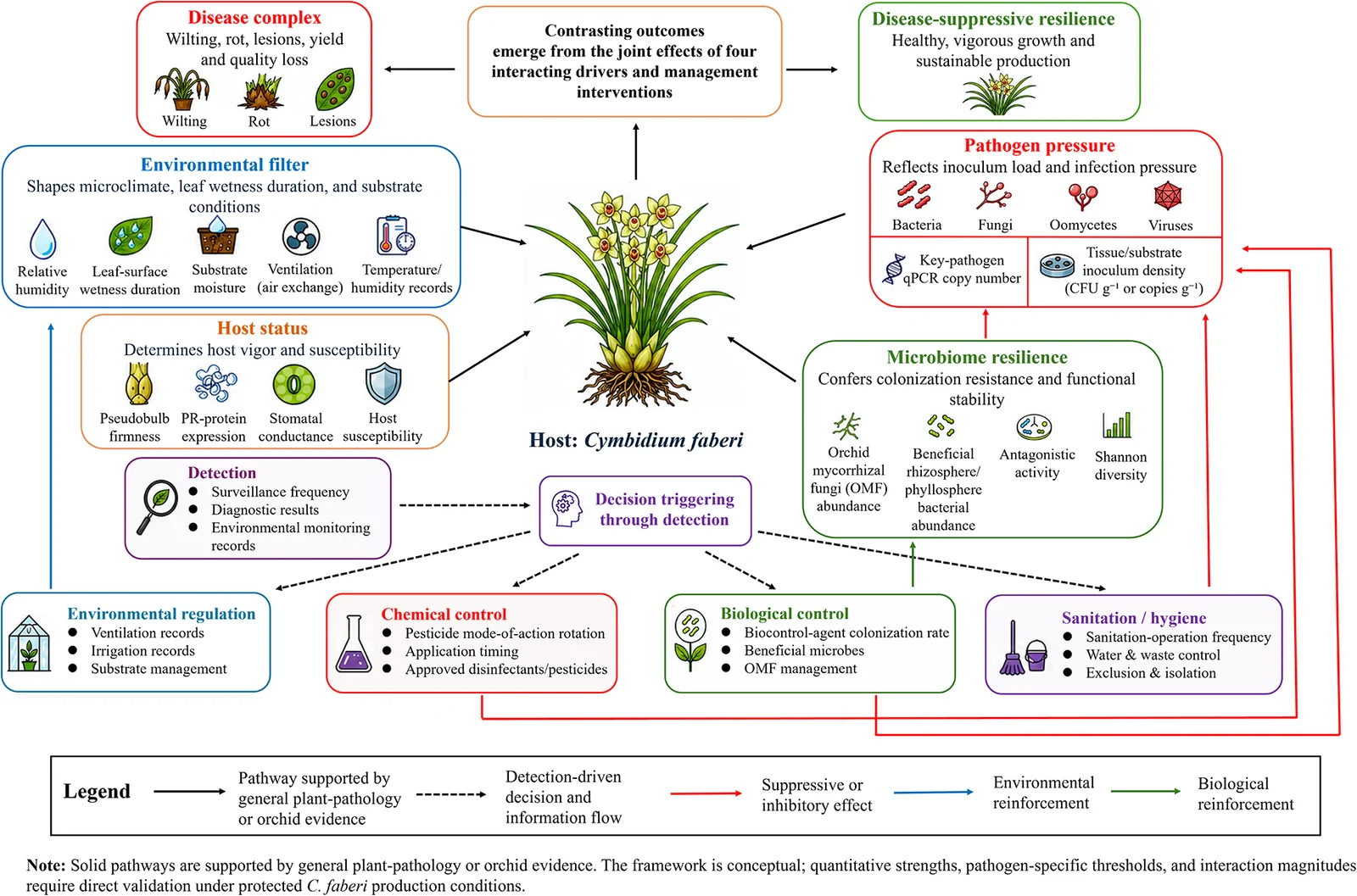
Conceptual model of interactions among environmental filtering, pathogen pressure, host status, microbiome resilience, and management interventions in protected *Cymbidium faberi* production. Environmental filtering, pathogen pressure, host status, and microbiome resilience jointly shape the system state of *C. faberi* and the balance between disease-complex development and disease-suppressive resilience. Representative measurable candidates are listed within each node. Detection results trigger management decisions involving environmental regulation, chemical control, biological control, and sanitation/hygiene. Black solid arrows indicate directional pathways supported by general plant-pathology or orchid evidence; black dashed arrows indicate detection-driven decision and information flow; red arrows indicate suppressive effects that reduce pathogen pressure; blue arrows indicate environmental regulation; and green arrows indicate biological reinforcement of microbiome resilience. The model is a translational conceptual framework; pathway strengths, pathogen-specific thresholds, indicator weights, and interaction magnitudes require direct validation under protected *C. faberi* production conditions. The listed indicators include both operational monitoring variables and research/validation metrics and should not be interpreted as being equally validated or equally ready for routine production deployment.

The model proposes that disease development or disease-suppressive resilience may emerge from interactions among environmental conditions, inoculum pressure, host vigor and susceptibility, microbial-community status, and management interventions rather than from a single pathogen-host relationship. Operationally, it supports three priorities: reducing prolonged humidity and leaf wetness, interrupting propagation-, tool-, and batch-mediated transmission, and integrating chemical suppression with biological, environmental, and hygiene measures. **Figure 2** should therefore be interpreted as a conceptual decision-support and validation framework, not as a quantitatively calibrated causal model.

## 6 Diagnosis and monitoring: a modular diagnostic pathway for protected production

Diagnostic methods differ in target dependence, analytical resolution, deployment setting, turnaround time, cost, and decision function (Ali et al., 2019; Venbrux et al., 2023). **Figure 3** therefore organizes them into four functional components: surveillance and anomaly prescreening, laboratory-based targeted testing, near-site targeted rapid testing, and untargeted discovery for unresolved or complex cases. Laboratory and near-site targeted tests are parallel options selected according to the management question, quantitation needs, infrastructure, and locally validated performance. A separate causal-confirmation branch is activated when direct pathogen-symptom attribution is required. This architecture structures method selection and escalation but is not a validated *C. faberi*-specific diagnostic standard.

**Figure 3:**
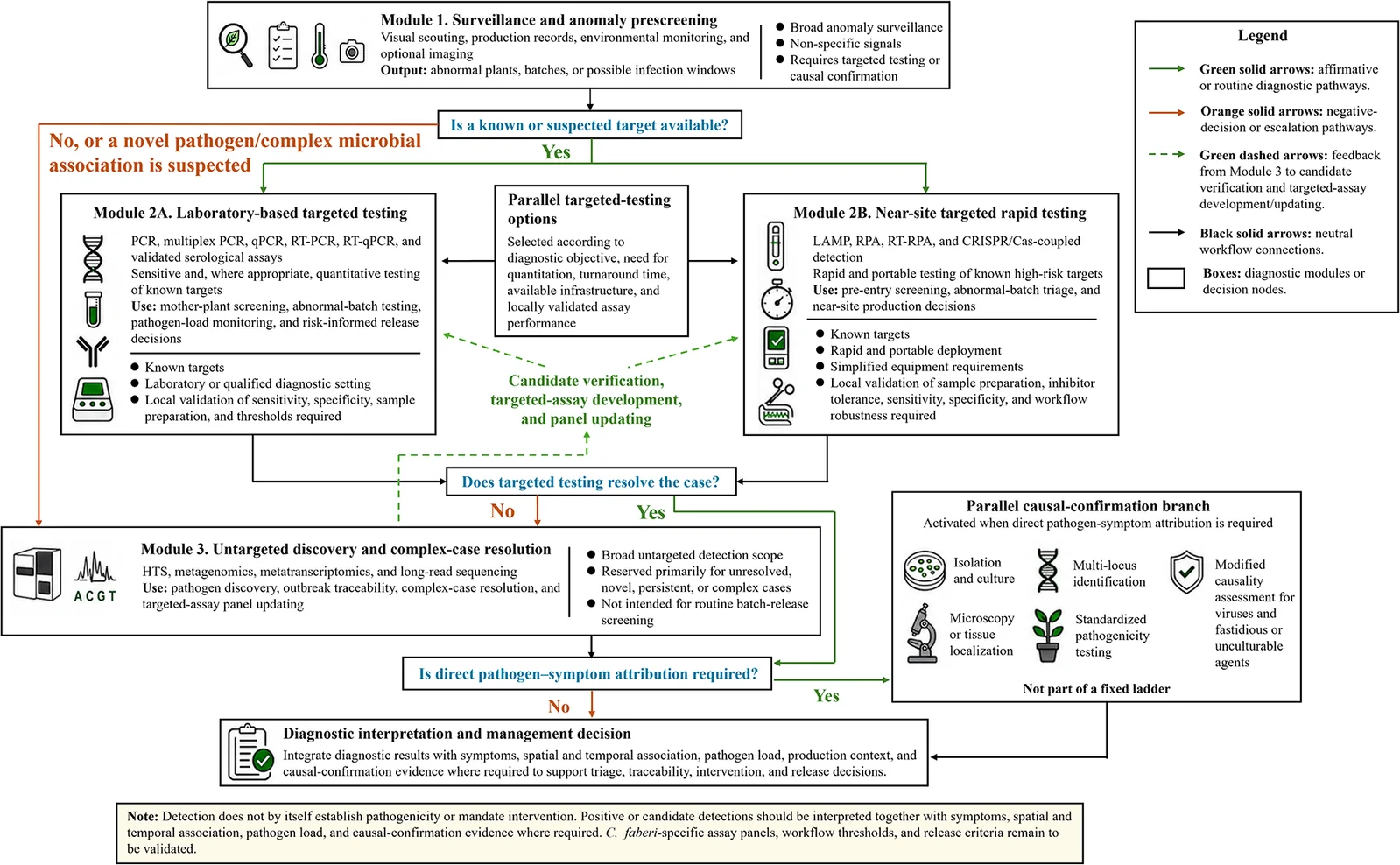
Modular diagnostic architecture for microbial disease risks in protected *Cymbidium faberi* production. The surveillance and anomaly-prescreening module identifies abnormal plants, batches, or possible infection windows. When a known or suspected target is available, laboratory-based targeted testing and near-site targeted rapid testing are used as parallel deployment options selected according to the diagnostic objective, need for quantitation, turnaround time, available infrastructure, and locally validated assay performance. Cases lacking a defined target, remaining unresolved after targeted testing, or suspected to involve a novel pathogen or complex microbial association are escalated to untargeted discovery and complex-case resolution. Outputs from untargeted analyses feed back into candidate verification, targeted-assay development, and panel updating. A separate causal-confirmation branch—including isolation and culture, microscopy or tissue localization, multi-locus identification, standardized pathogenicity testing, and modified causality assessment for viruses and fastidious or unculturable agents—is activated when direct pathogen-symptom attribution is required. Green solid, orange solid, green dashed, and black solid arrows indicate affirmative or routine pathways, negative-decision or escalation pathways, feedback pathways, and neutral workflow connections, respectively. Boxes indicate functional diagnostic modules or decision nodes. The architecture is a translational decision-support framework rather than a validated *C. faberi*-specific diagnostic standard.

Module 1: Surveillance and anomaly prescreening. This module provides broad anomaly surveillance through visual scouting, production records, environmental monitoring, and, where technically and economically feasible, imaging technologies. Visual symptoms, greenhouse temperature and humidity, leaf-wetness duration, and substrate-moisture records can identify abnormal batches and possible infection windows. Hyperspectral, thermal, or chlorophyll-fluorescence imaging may support earlier physiological anomaly detection, but these technologies vary substantially in cost and production readiness (Walsh et al., 2024). Signals generated by this module are non-specific and require targeted testing or causal confirmation because water deficit, nutrient imbalance, salt injury, pest damage, and mechanical injury may produce similar responses (Shafay et al., 2025).

Module 2A: Laboratory-based targeted testing. This module is used for sensitive and, where appropriate, quantitative detection of known target pathogens under laboratory or qualified diagnostic conditions. Conventional PCR, multiplex PCR, qPCR, RT-PCR, RT-qPCR, and validated serological assays can support mother-plant screening, abnormal-batch testing, pathogen-load monitoring, and risk-informed release decisions (Venbrux et al., 2023; Kanapiya et al., 2024). Published assays developed for other *Cymbidium* or orchid hosts provide methodological starting points, but primer or probe performance, sample preparation, analytical sensitivity, specificity, and decision thresholds require *C. faberi*-specific or local validation before routine batch-release use. For batch-based screening of orchid asexual propagation material, Eun et al. (2000) established a TaqMan real-time RT-PCR assay for the simultaneous quantitation of CymMV and ORSV. At the regional Chinese *Cymbidium* level, Ren et al. (2020) combined DAS-ELISA with RT-PCR to test symptomatic production samples for CymMV and ORSV, illustrating a two-tier screening-and-confirmation workflow that is operationally closer to Chinese *Cymbidium* production than evidence derived exclusively from *Phalaenopsis* or other commercial orchids. However, symptom-based sampling and the absence of *C. faberi*-resolved results preclude the use of these data as species-specific prevalence estimates or batch-release thresholds. Serological assays such as ELISA can support high-throughput preliminary screening of these viruses where validated antibodies are available, although their analytical sensitivity is generally lower than that of nucleic acid assays. The multiplex PCR established by Kim et al. (2024) for *C. kanran* simultaneously detects *Colletotrichum gloeosporioides*, *Pestalotiopsis* sp., *F. oxysporum*, and *F. proliferatum*, providing a methodological reference for multi-pathogen screening of *Cymbidium* foliage. These methods were developed primarily using *C. kanran*, *Phalaenopsis*, or other orchid systems; *C. faberi*-specific multi-pathogen detection panels and batch-release thresholds remain to be established.

Module 2B: Near-site targeted rapid testing. This module provides a field-deployable or near-site alternative for rapid detection of known high-risk targets. Isothermal amplification methods, including LAMP, RPA, and RT-RPA (Gomez-Gutierrez and Goodwin, 2022; Panno et al., 2020), and CRISPR/Cas-coupled detection systems (Aman et al., 2020) may shorten turnaround time and support pre-entry screening, abnormal-batch triage, and regional production decisions. These approaches are not inherently more specific or analytically superior to laboratory-based PCR or qPCR; their main advantage lies in portability, simplified equipment requirements, and faster deployment. Selection between Module 2A and Module 2B should therefore depend on the diagnostic objective, required quantitation, available infrastructure, turnaround time, and locally validated assay performance. Sun et al. (2024) developed a duplex RT-RPA rapid assay for the simultaneous detection of CymMV and ORSV, with potential application in pre-entry seedling screening after local validation. Lee et al. (2024) combined Nanopore sequencing with RT-RPA-CRISPR/Cas12a detection in *Phalaenopsis* virus identification, providing a methodological reference for rapid orchid virus diagnosis. However, these systems currently mainly serve commercial orchids such as *Phalaenopsis*; sample pretreatment, inhibitor tolerance, analytical sensitivity, false-positive control, and workflow robustness require dedicated validation in *C. faberi* tissues before routine near-site deployment.

Module 3: Untargeted discovery and complex-case resolution. This module is activated when targeted tests are negative despite persistent disease, symptoms remain difficult to attribute, a novel virus or pathogen variant is suspected, or complex microbial associations require investigation (Nizamani et al., 2023; Maree et al., 2018; Villamor et al., 2019). High-throughput sequencing (HTS), metagenomics, metatranscriptomics, and long-read sequencing can broaden rather than narrow detection scope because they do not require a predefined target (Massart et al., 2017). Their primary role is pathogen discovery, outbreak traceability, complex-case resolution, and assay-panel updating rather than routine production-side batch release. Candidate agents identified through untargeted methods should subsequently be evaluated using targeted assays, spatial or temporal association, and, where biologically feasible, causal-confirmation procedures.

Parallel causal-confirmation branch: isolation, culture, histological localization, and pathogenicity testing. The causal-confirmation branch is functionally distinct from surveillance and routine detection. It should be activated when a detected organism must be linked directly to symptoms, when abnormal batches require etiological traceability, when candidate biocontrol strains or antimicrobial responses are evaluated, or when co-infection and sequential-infection mechanisms are investigated. Isolation and culture, microscopy or tissue sectioning, multi-locus identification, and standardized pathogenicity testing following Koch’s postulates (Bhunjun et al., 2021) can support causal attribution. For viruses, fastidious organisms, or unculturable agents, modified causality frameworks combining molecular detection, localization, transmission evidence, and reproducible symptom association may be required.

### 6.1 Methodological limitations and transferability boundaries

Method selection should be driven by the management question rather than by an assumed linear hierarchy of technological sophistication. When translating the above diagnostic technologies to *C. faberi* production, a distinction must be made between “detectable” and “pathogenic.” Polysaccharides, polyphenols, and secondary metabolites in leaves and pseudobulbs may inhibit amplification-based assays, so extraction and inhibitor-tolerance performance should be verified locally. The quantitative capability of qPCR/RT-qPCR depends on standard curves, internal-reference selection, and sample homogenization; threshold settings may lack comparability between laboratories. Therefore, molecular-detection schemes should prioritize primers and probes that have been validated in published studies or locally, and combine isolation and culture, pathogenicity testing, and symptom tracking for cross-confirmation, avoiding equating detection directly with pathogenicity or mandatory intervention (Venbrux et al., 2023).

## 7 Disease management toolbox

*C. faberi* microbial disease management should be viewed as a set of measures that can be combined flexibly by scenario; its effectiveness depends on the linkage of measures across stages and the consistency of actual implementation (Daughtrey and Buitenhuis, 2020; Buirs and Punja, 2024). Direct *C. faberi* disease-management research is extremely limited. Among the 82 scientific publications summarized in **Supplementary Table S2**, one primary study directly addressed *C. faberi* disease management by screening plant extracts *in vitro* against an anthracnose-associated isolate obtained from *C. faberi* (Liu et al., 2009). One additional study isolated candidate antagonistic endophytes from *C. faberi* but evaluated *in vivo* suppression in *C. goeringii* (Jiang et al., 2023). The count above refers specifically to direct-host disease-management studies and does not represent all management-related publications from other orchids, protected crops, or general plant-pathology systems used elsewhere in this section. The remainder of this section therefore mainly draws on three categories of translatable evidence: virus rapid detection and virus-free propagation from commercial-orchid studies; oomycete and soft-rot management from orchid *Phytophthora*- and *Pectobacterium*-related studies; and greenhouse microclimate control, zoning hygiene, and mode of action (MoA) rotation from ornamental-plant disease-management experience. Such evidence is transferred to *C. faberi* only where pathogen characteristics, transmission media, and production stages are sufficiently comparable, with extrapolation boundaries stated where cultivar, environment, or formulation conditions differ.

### 7.1 Prevention and cultural management

Preventive measures are likely to form the foundation of *C. faberi* disease-risk management because many have broad cross-disease applicability and potentially high operational value, although their effectiveness and cost-benefit performance require local evaluation; this principle is widely supported in greenhouse ornamental and nursery systems (Guarnaccia et al., 2021; Daughtrey and Buitenhuis, 2020). Given *C. faberi*’s dependence on asexual propagation and batch turnover, and consistent with the general principles of plant virus latency (Takahashi et al., 2019), latent and asymptomatic virus infections markedly increase the risk of missed detection when relying solely on visual screening; drawing on high-sensitivity virus detection methods developed for commercial orchids (Eun et al., 2000), molecular testing should be combined with the removal of infected material and locally validated clean-propagation procedures to reduce the risk of infected mother plants entering the protected-production chain. At the operational level, referring to general practices of tool sterilization in crops such as banana and zoning management of greenhouse ornamental plants (Ocimati et al., 2021; Daughtrey and Buitenhuis, 2020), standardized tool disinfection, zoning and grading, and batch-isolation systems should be evaluated, locally validated, and progressively implemented to reduce mechanical transmission. At the environmental and media level, referring to reports on pathogen monitoring of nursery return water and water-borne spread of orchid *Phytophthora* (Redekar et al., 2020; Bag et al., 2024), hygiene management of water sources, piping, and return-water systems should be incorporated into routine control to reduce the recurrent input risk of water-borne pathogens such as oomycetes.

Virus-elimination protocols established in commercial orchids, including meristem-tip culture with or without thermotherapy, provide a basis for developing clean-stock programs in *C. faberi*. Such programs should include locally validated retesting intervals for mother-plant collections. Substrate composition (e.g., bark-versus sphagnum-based mixes), irrigation mode (overhead versus sub-irrigation), and pot spacing should also be treated as risk-modifying covariates because they shape canopy humidity, leaf-wetness duration, and splash dispersal. The above preventive strategies mostly derive from mature experience with greenhouse ornamental plants and major crops; their applicability, threshold parameters, and localized standards in protected *C. faberi* production require targeted validation through epidemiological studies under protected-production conditions and economic evaluation.

### 7.2 Chemical control

In protected ornamental plant production, chemical control remains practically relevant and is mainly used for protective treatment and early suppression of diseases such as leaf spot, anthracnose, and gray mold/flower rot (Guarnaccia et al., 2021). Its efficacy highly depends on application timing and coverage quality; when disease has progressed to extensive tissue necrosis or rapid expansion of bacterial soft rot, chemical agents alone may be insufficient to reverse disease progression, and isolation-and-removal, dehumidification and ventilation, and transmission-chain blockade should proceed in parallel (Charkowski, 2018). Long-term high-frequency application may increase resistance-selection pressure; therefore, MoA rotation should be implemented according to registration requirements and pathogen fungicide sensitivity, and chemical measures embedded within the IDM framework (Corkley et al., 2022). In addition, the disturbance of non-target phyllosphere/rhizosphere microbial communities by pesticides may disrupt disease-suppressive networks, bringing an ecological trade-off of short-term suppression followed by recurrence; agents should therefore be positioned as suppression tools for key windows rather than as the sole pillar (Sangiorgio et al., 2022). A localized *C. faberi* study also screened nine plant extracts against an anthracnose-associated isolate under *in vitro* conditions (Liu et al., 2009). This provides preliminary direct-host antimicrobial-screening evidence, but plate-based inhibition does not establish *in planta* efficacy, phytotoxicity, formulation stability, effective dose, or regulatory suitability under protected production. Beyond this localized screening report, systematic *C. faberi* research on registered disease-control products, pathogen-specific sensitivity baselines, resistance monitoring, mode-of-action rotation, and pesticide effects on phyllosphere and rhizosphere microbiomes under protected-cultivation conditions remains lacking.

### 7.3 Biological control and immune induction

Biological control can contribute to sustainable disease management through two pathways: direct disease suppression by antagonistic microbes and enhancement of host defense by growth-promoting or resistance-inducing endophytic fungi (Fontana et al., 2021; Bonaterra et al., 2022). Regarding direct-host evidence, Jiang et al. (2023) isolated six endophytic fungi from healthy root segments of *C. faberi* ‘Hongxiangfei’. *Fusarium solani* strain Z2 inhibited *F. oxysporum in vitro* by 67.51%, and *Dichotomopilus funicola* strain Z3 inhibited *Colletotrichum gloeosporioides* by 68.56%. Z3 treatment subsequently reduced diseased-leaf rate and disease index in an *in vivo* test using *C. goeringii* ‘Green Cloud’, rather than *C. faberi*. The study therefore provides *C. faberi*-derived candidate endophytes and cross-species *in vivo* proof of concept, but not protected-production efficacy in *C. faberi*. Wen et al. (2025) also isolated root endophytic fungi from wild *C. faberi* and *C. goeringii* populations and identified strains with growth-promoting traits; such traits should not be equated with disease suppression. Before formulation development or protected-production testing, candidate endophytic strains—particularly isolates belonging to taxa that include plant-pathogenic lineages—should undergo taxonomic re-identification, pathogenicity and host-range testing, metabolite or toxin assessment, and non-target biosafety evaluation. *In vitro* antagonism, growth-promoting traits, and cross-species pot-level suppression should therefore be treated as sequential screening evidence rather than as proof of production suitability in *C. faberi*. In other *Cymbidium* species and protected crops, the bottleneck from effective strain to stable product often lies in scale-up production, formulation engineering (carriers, viable-count maintenance, shelf life), and the adaptation of application nodes to the production workflow (Teixidó et al., 2022). Regarding induced resistance (SAR/ISR) and chemical/biological elicitors, available mechanistic and experimental evidence is derived mainly from model plants and major crops; reproducible data in orchids are insufficient, and these are better positioned as preventive-gain measures before risk windows, with plot/batch trials conducted first (Hönig et al., 2023). Accordingly, strain-cultivar-environment matrix trials and formulation-process research are the main priorities.

### 7.4 Physical exclusion and ecological regulation

Physical and ecological regulation mainly target inoculum accumulation and transmission media. Heat/steam treatment of substrates and seedling containers can reduce pathogen loads before planting, but efficacy depends on treatment intensity and subsequent recontamination control (Gullino et al., 2022). At the irrigation-water or nutrient-solution stage, UV disinfection has some potential against water-borne oomycetes, but efficiency is affected by dose, water turbidity, organic matter, and hydraulic residence time, making it better suited as a component module of a water-treatment system (Ghimire et al., 2024). Substrate amendment and the development of disease-suppressive substrate microbiomes can promote the establishment of beneficial communities, but this process requires time to accumulate and is easily reset by repeated stringent disinfection; it should be implemented in coordination with biological control and nutrient management (Spooren et al., 2024). In *C. faberi*, direct validation of substrate heat treatment, UV water disinfection, and disease-suppressive substrate-microbiome development is still lacking. The deliberate establishment of disease-suppressive microbiomes in protected orchid substrates has received little direct investigation. Protected-production trials are therefore needed to evaluate substrate-microbiome co-management in *C. faberi*.

### 7.5 Disease-resistance breeding and biotechnology

Disease-resistance breeding and biotechnology represent medium- to long-term options for *C. faberi* disease management. Experience in ornamental orchids such as *Phalaenopsis* indicates that long breeding cycles and simultaneous selection for ornamental and growth traits can limit the rapid deployment of resistant cultivars (Iiyama et al., 2024). Resistance phenotypes for complex diseases are influenced by environmental conditions, plant physiological state, inoculum load, and scoring methods; standardized inoculation and phenotyping procedures and cross-environment validation are therefore needed (Possamai and Wiedemann-Merdinoglu, 2022). Marker-assisted selection (MAS) can facilitate the tracking and pyramiding of resistance loci and may shorten the breeding cycle (Hasan et al., 2021). RNA interference (RNAi), exogenous double-stranded RNA (dsRNA), and related approaches represent potential strategies for targeted disease-resistance improvement, but effective delivery, field stability, and consistency remain engineering bottlenecks; regulatory differences and public perception will also affect the pace of industrialization (Venu et al., 2025; Spök et al., 2022). *C. faberi*-specific information on disease-resistance germplasm, molecular markers, and targeted biotechnology remains limited; stronger resistance-germplasm evaluation and genomic-tool development are therefore needed for medium- to long-term improvement.

## 8 Integrated disease management: a translational decision framework by production nodes

We propose a node-based translational IDM framework for protected *C. faberi* production. The framework is not a fixed technology checklist or validated production standard; rather, it assigns detection, prevention, intervention, and clearance responsibilities to specific production nodes (**Table 2**) using limited CF evidence together with translatable CY, OR, and GH evidence. The chain can be decomposed into seven nodes: mother-plant accession, TC/clonal-division propagation, acclimation and potting, vegetative growth, flowering management, abnormal-batch disposition, and release/shipping clearance (Buirs and Punja, 2024; Daughtrey and Buitenhuis, 2020). The resulting node-based IDM sequence is summarized in the decision-flow framework (**Figure 4**).

**Table 2:** Node-based decision points for IDM in *C. faberi* protected production.

Production node	Core responsibility	Key tools/detection
Mother-plant accession	Screen for and exclude virus-infected or systemic-pathogen-positive material	Published CymMV/ORSV RT-qPCR or RT-PCR assays followed by *C. faberi*-specific or local validation; accession and source records
TC/clonal-division propagation	Prevent mechanical transmission through tool sanitation, batch separation, and traceable propagation records	Locally validated disinfectants; tool-treatment records, batch identity and isolation records
Acclimation and potting	Maintain substrate aeration and reduce substrate- and irrigation-borne inoculum	Locally qualified substrate sanitation or treatment; irrigation-water monitoring and treatment where required
Vegetative growth	Shorten periods of excessive humidity, prolonged leaf wetness, and over-wet substrate conditions	Ventilation, irrigation scheduling, plant spacing, shading, and environmental monitoring
Flowering management	Senescent-flower removal and peri-anthesis microclimate	Dehumidification, senescent-flower removal
Abnormal-batch disposition	Select targeted testing, untargeted discovery, or causal confirmation according to symptom pattern, risk context, and decision need	Modular diagnostic pathway; laboratory or near-site targeted tests; untargeted sequencing for unresolved cases; causal-confirmation branch when required
Release/shipping clearance	Apply provisional release criteria based on locally validated or qualified target-specific negative tests, absence of relevant symptoms, and complete batch records	Locally validated or qualified target-specific testing; symptom inspection and batch traceability records

Each node pairs a core responsibility with the key tools/detection methods to be applied at that production stage; the seven-node sequence is visualized as a decision flow in **Figure 4**. The table operationalizes the translational IDM framework and is based primarily on CY, OR, and GH evidence, with MP evidence supporting general diagnostic and management principles. All node-specific thresholds, retesting intervals, and release criteria require *C. faberi*-specific validation. The diagnostic pathway is modular rather than strictly linear; laboratory-based and near-site targeted tests are parallel deployment options selected according to the management question and available infrastructure.

**Figure 4:**
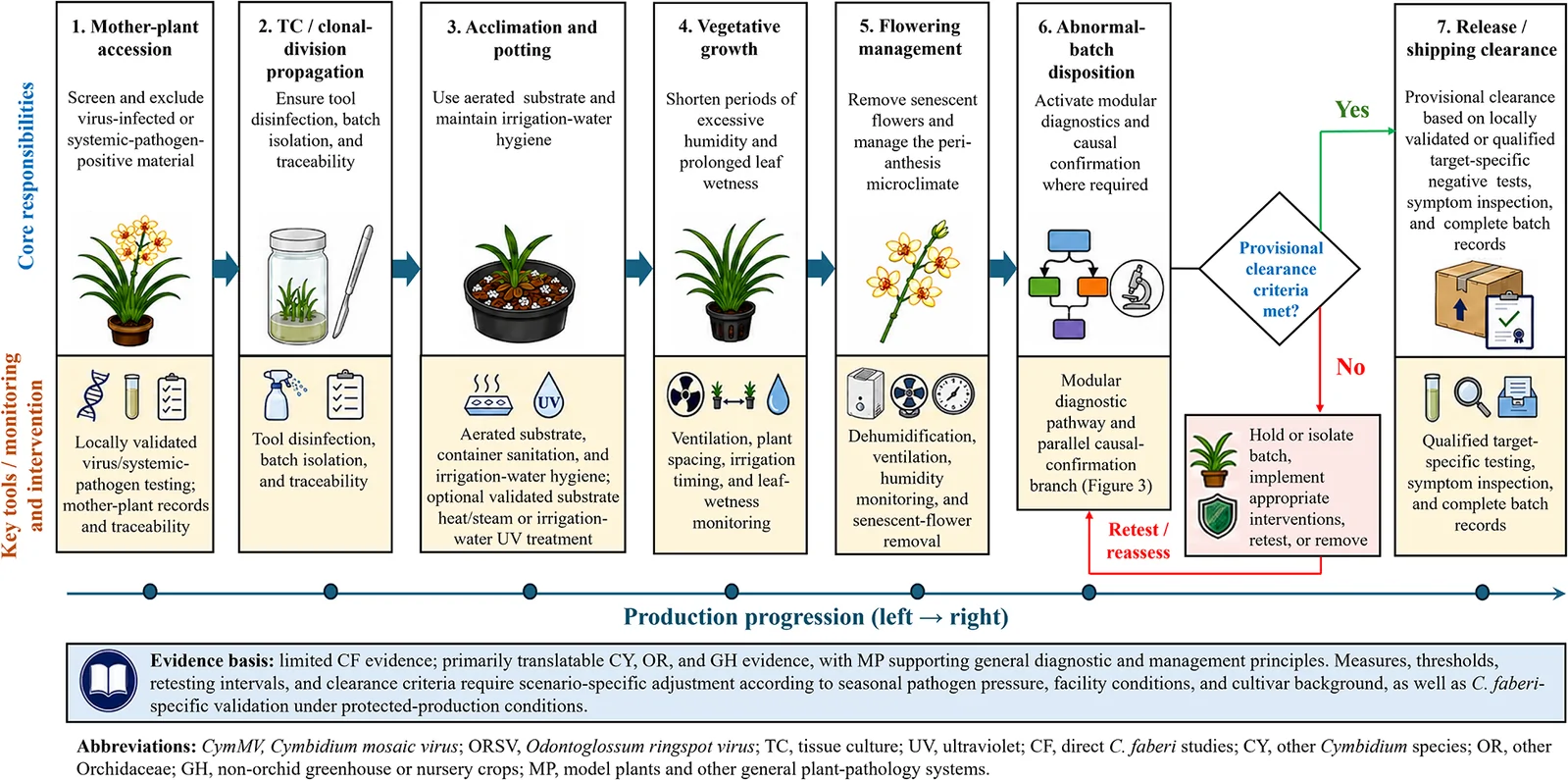
Seven-node decision flow of Integrated Disease Management (IDM) for protected *Cymbidium faberi* production. Detection, prevention, monitoring, intervention, and clearance responsibilities are assigned across seven production nodes: mother-plant accession, tissue-culture or clonal-division propagation, acclimation and potting, vegetative growth, flowering management, abnormal-batch disposition, and release or shipping clearance. Here, an “abnormal batch” denotes a batch showing atypical symptoms, abnormal growth, or monitoring signals that trigger diagnostic escalation; it is a decision category rather than a diagnosis. The upper sections summarize the core responsibilities of each node, whereas the yellow lower sections show representative tools, monitoring measures, and intervention options. Abnormal batches activate the modular diagnostic pathway and, where direct etiological attribution is required, the parallel causal-confirmation branch shown in **Figure 3**. Batches meeting provisional clearance criteria proceed to release or shipping; those failing to meet the criteria are held or isolated for intervention, retesting, reassessment, or removal. Blue arrows indicate production progression, the green arrow indicates conditional clearance, and red arrows indicate non-clearance and feedback. The framework is a provisional decision-support structure based on limited direct *C. faberi* evidence and translatable orchid, protected-crop, and general plant-pathology evidence; its measures, thresholds, retesting intervals, and clearance criteria require scenario-specific adjustment and *C. faberi*-specific validation.

The core responsibilities of each node can be summarized as follows: mother-plant accession focuses on screening and excluding virus-infected or systemic-pathogen-positive material; TC/clonal-division propagation focuses on tool disinfection and batch isolation; acclimation and potting focus on substrate aeration and irrigation hygiene; the vegetative-growth stage focuses on shortening periods of excessive humidity and prolonged leaf wetness; flowering management focuses on senescent-flower removal and peri-anthesis microclimate; abnormal-batch disposition activates the modular diagnostic pathway and, where direct etiological attribution is required, the parallel causal-confirmation branch described in Section 6; and release/shipping clearance uses provisional release criteria based on locally validated or qualified target-specific negative tests, symptom inspection, and complete batch records. Specific measure combinations are provided in **Supplementary Table S1** and can be dynamically adjusted according to seasonal pathogen pressure, facility conditions, and cultivar background. The framework is intended to embed disease-risk monitoring and management responsibilities within the protected-production pipeline and to provide an operational structure for subsequent *C. faberi*-specific calibration and protected-production testing.

## 9 Discussion and perspectives

### 9.1 Key controversies and evidence boundaries

Although substantial information has accumulated on pathogen identification and management technologies, translation from the current evidence base to a *C. faberi*-specific management system remains constrained by three core controversies.

Controversy 1: Is co-infection synergistic pathogenesis or simple superposition? Multi-pathogen coexistence has been repeatedly reported in existing *Cymbidium* and related orchid disease systems, but its incidence, stability, and the question of “whether coexistence equals synergistic pathogenesis” in plants still highly depend on experimental design. *Burkholderia gladioli* isolated from leaves by Moon et al. (2017) can infect alone, whereas the pseudobulb rot reported by Sen et al. (2006) was jointly caused by bacteria, *Fusarium*, and saprophytic fungi, suggesting that different combinations may present a continuous spectrum from coexistence to sequential pathogenesis. The key limitation is that most available studies demonstrate coexistence through isolation or molecular detection without factorial experiments controlling inoculum load, infection sequence, and cultivar background. We therefore propose environmental stress followed by opportunistic or sequential colonization as a testable working hypothesis for protected *C. faberi* production, rather than as an established infection sequence. Directly equating multi-pathogen detection with synergistic pathogenesis could lead to premature assumptions about pathogen-combination-specific interventions. The hypothesis is informed by reports from related *Cymbidium* systems and general concepts of multi-pathogen interactions, but remains to be tested through controlled single-, co-, and sequential-inoculation experiments in *C. faberi*.

Controversy 2: Can OMF-mediated disease suppression translate from controlled experiments to protected production? Evidence that OMF can induce ISR in specific orchid systems mainly derives from controlled pot experiments (Pujasatria et al., 2024). Direct *C. faberi* studies have documented environmental and geographic differentiation in root-endophytic and rhizosphere microbial assemblages and have recovered candidate mycorrhizal or beneficial taxa, including *Tulasnella* (Wang et al., 2015; Yu et al., 2015; Luo et al., 2019; Lv et al., 2023a; Du et al., 2025; Wen et al., 2025). However, none has tested whether these communities, defined OMF taxa, or candidate strains reproducibly suppress disease under protected *C. faberi* production. The central translational gap is therefore functional validation and production stability, rather than the complete absence of direct-host microbiome information. The key contradiction is that substrate replacement, agrochemical use, high temperature and humidity, and repeated vegetative division during clonal propagation may weaken or reshape OMF communities, whereas experimental systems and commercial protected production may differ markedly in total inoculum density, the relative inoculum ratios of interacting microbial or pathogen strains, and the physiological and environmental stress background of the orchid host. Accordingly, OMF-mediated disease suppression is biologically plausible in *C. faberi* but should not currently be adopted as a core component of protected-production management. A more prudent approach is to treat it as a long-term component of substrate-microbiome management and to evaluate colonization stability, pesticide compatibility, and disease-suppressive performance through multi-location and multi-season trials. The biological rationale is derived mainly from other orchid systems, and its performance under protected *C. faberi* production remains unknown.

Controversy 3: What are the dominant drivers of variation in biological-control efficacy? Endophytic fungi isolated by Jiang et al. (2023) from *C. faberi* roots showed relatively high inhibition rates against *F. oxysporum* and *Colletotrichum gloeosporioides in vitro*, and one candidate reduced anthracnose symptoms in *C. goeringii*. Nevertheless, a substantial gap remains between these screening results and reproducible efficacy in protected *C. faberi* production. The key contradiction is that existing literature often reports “effective strains” in isolation, making it difficult to separate the relative contributions of strain-host-pathogen-genotype interactions, protected-environment filtering, and formulation engineering. In the absence of *C. faberi*-specific production trials, biological control should currently be positioned as a preventive supplement before high-risk windows rather than as an independent replacement for chemical control. Future studies should use strain-cultivar-pathogen-environment matrix designs and formulation-process experiments to identify the dominant sources of efficacy variation. The available direct *C. faberi* information comprises *in vitro* antagonistic activity and cross-species *in vivo* proof of concept, and stable efficacy under protected-production conditions remains to be established.

### 9.2 Validation agenda and priority research directions

To support the transition from a translational framework to a validated *C. faberi*-specific management system, we organized the principal evidence gaps into a prioritized validation agenda. We ordered these priorities according to the extent to which each unresolved issue constrains pathogen attribution, risk-threshold calibration, intervention selection, or production-node decision-making. The corresponding knowledge gaps, study designs, and expected management outputs are summarized in **Table 3**.

**Table 3:** Validation agenda and priority research directions for protected *Cymbidium faberi* production.

Priority	Key knowledge gap	Main evidence gap	Recommended study design	Expected management output
First priority	*C. faberi* candidate pathogen inventory, causal validation, and detection-panel development	Direct *C. faberi* evidence includes baseline studies of root endophytes and rhizosphere microbial communities together with one localized anthracnose-oriented pathogen-identification report. Chinese *Cymbidium*-level virus surveys are available but are not resolved specifically to *C. faberi*. A contemporary, multi-region, causally supported *C. faberi* pathogen inventory and a validated disease-detection panel for protected production remain lacking.	Systematically sample symptomatic *C. faberi* across representative production regions, including anthracnose-like lesions from Luanchuan and other major production areas. Re-isolate and identify candidate pathogens using morphology, multilocus sequencing or phylogenomic methods, and standardized pathogenicity testing. Conduct virus surveys resolved by *C. faberi* cultivar, propagation source, production stage, and symptom status. On this basis, develop and validate multiplex qPCR/RT-qPCR detection panels and risk-informed batch-release thresholds.	Establish an initial causally supported *C. faberi* pathogen inventory, provisional detection panels, and risk-informed release criteria for subsequent multi-site validation
Second priority	Greenhouse microclimate–substrate–disease risk thresholds and node-based IDM protected-production validation	Environmental thresholds and node-based management measures are derived largely from other greenhouse crops; *C. faberi*-specific temperature, humidity, leaf-wetness, and substrate-moisture parameters remain poorly defined.	Conduct multi-location, multi-season protected-cultivation trials coupling environmental sensors (temperature, humidity, leaf wetness, substrate moisture) with disease incidence recorded at each IDM node	Empirically calibrate *C. faberi*-specific temperature–humidity–substrate-moisture risk thresholds and deliver a production-tested prototype of the node-based IDM protocol
Third priority	Temporal infection sequences and co-infection mechanisms of priority candidate pathogens confirmed through first-stage causal studies	Multi-pathogen coexistence and interaction concepts are reported in related orchid and general plant-pathology systems, but *C. faberi*-specific infection sequences, interaction effects, and synergistic mechanisms remain unvalidated.	After single-pathogen causality has been established for the strains concerned, systematically compare selected factorial co-inoculation treatments involving priority soft-rot bacteria, oomycetes, and *Fusarium*, followed by sequential-inoculation experiments where biologically justified, with inoculum-load control, repeated temporal sampling, and cultivar-background replication	Identify pathogen combinations or temporal sequences that reproducibly modify symptom severity, disease progression, or intervention urgency, providing an experimentally supported basis for abnormal-batch disposition
Fourth priority	Protected-production stability of microbiome regulation (OMF/biocontrol strains)	Direct *C. faberi* studies provide baseline information on culturable root-endophytic bacteria and fungi, rhizosphere bacterial and fungal communities, and candidate antagonistic endophytes. Root-associated fungi, including *Tulasnella*, have been recovered from *C. faberi*, but their mycorrhizal status, *in planta* colonization stability, disease-suppressive function, formulation performance, pesticide compatibility, and production-scale efficacy remain unvalidated. The *in vivo* biocontrol test reported by Jiang et al. (2023) used *C. goeringii* rather than *C. faberi*.	Select representative *C. faberi* cultivars and production-region substrates for multi-location, multi-season protected-production trials that combine longitudinal root/rhizosphere community profiling with taxonomic confirmation and microscopy- or qPCR-based colonization tracking of candidate OMF or antagonistic strains, standardized disease challenge, disease incidence and severity assessment, and compatibility testing with routine pesticides.	Identify microbial strains or communities that reproducibly colonize *C. faberi* and suppress validated priority diseases, and define production-stage application windows, formulation requirements, and pesticide-compatibility schemes.
Fifth priority	*C. faberi* immune-genetic basis	A sufficiently complete and functionally annotated *C. faberi* reference genome and species-specific immune-genomic analyses are currently lacking; interpretation therefore relies on comparative orchid genomics and general plant-immunity studies.	Use genomics/transcriptomics to characterize NLR and other defense-associated gene families, defense-metabolism trade-offs, and virus–fungus–bacteria cross-immune-signaling networks	Provide targets for MAS and disease-resistance breeding
Sixth priority	Multi-source-data-integrated disease prediction models	Disease-prediction approaches are well developed in other greenhouse and crop systems, but parameterized, prospective *C. faberi* production data are lacking.	Integrate environmental sensors, imaging phenotypes, and molecular-detection data to build data-driven early-warning and decision-support systems, and validate them prospectively in typical production regions	Achieve production-node-based risk early warning and decision-support recommendations for risk-adapted and minimally sufficient intervention combinations

Priorities are ordered by the degree of constraint on management decisions; the corresponding evidence controversies are discussed in Section 9.1.

The realization of the above research depends on multidisciplinary collaboration across plant pathology, microbial ecology, agricultural engineering, and information science. Multi-omics integration can provide a systems-biology perspective for complex interactions (Balotf et al., 2025; Murmu et al., 2024), whereas drawing on the modeling principles of plant disease prediction models and conducting prospective validation in production regions is key to enhancing early-warning capability (González-Domínguez et al., 2023; Hasanaliyeva et al., 2022). Only by fully acknowledging evidence limitations and filling key knowledge gaps can *C. faberi* management measures progressively transition from a translatable framework to practical solutions that withstand protected-production validation (Leveau, 2024; Soubeyrand et al., 2024). Together, these priorities define the empirical pathway required to convert the proposed translational framework into a pathogen-validated, threshold-calibrated, and production-tested IDM system, providing scientific and technological support for the sustainable development of the East Asian *Cymbidium* orchid industry.

### 9.3 Limitations of this review

Several limitations should be considered when interpreting this review. First, this article is a critical narrative review rather than a systematic review, meta-analysis, or exhaustive bibliographic assessment. The literature cited was used to address the principal biological, diagnostic, and management questions considered in the review and should not be interpreted as a complete corpus of all publications related to *C. faberi* or orchid diseases. Second, direct *C. faberi* evidence includes studies of root-endophytic bacterial and fungal diversity, rhizosphere-community characterization, candidate antagonistic endophytes, and one localized anthracnose-oriented pathogen-identification and plant-extract-screening report. A regional survey additionally provides Chinese *Cymbidium*-level evidence for CymMV and ORSV, but the results were not resolved specifically for *C. faberi*. These studies do not constitute a contemporary multi-region pathogen inventory and remain insufficient to establish species-specific epidemiology, disease-associated microbiome dynamics, management thresholds, or stable intervention efficacy under protected production. Many interpretations concerning candidate pathogens, infection mechanisms, diagnostic approaches, and management options therefore continue to rely on studies of related *Cymbidium* species, other orchids, greenhouse crops, or general plant-pathology systems. Third, the CF, CY, OR, GH, and MP labels are descriptive indicators of the original study context rather than formal quality or evidence grades. Finally, many proposed management components lack species-specific thresholds, production-scale testing, or prospective validation. The conceptual model, modular diagnostic pathway, and node-based IDM framework should therefore be regarded as an author-derived translational synthesis and a set of testable management hypotheses rather than formal evidence-based guidelines for *C. faberi* production.

## 10 Conclusion

Three core judgments support the translational framework and validation agenda proposed in this review. First, direct *C. faberi* evidence comprises baseline information on root-associated bacterial and fungal diversity, rhizosphere microbial communities, candidate endophytes, and one localized anthracnose-oriented pathogen-identification and *in vitro* plant-extract-screening report. Broader Chinese *Cymbidium* evidence also documents the occurrence of CymMV and ORSV in symptomatic production samples. However, a contemporary and causally supported *C. faberi* pathogen inventory, species-resolved virus epidemiology, disease-associated microbiome dynamics, and validated protected-production management efficacy remain limited. Current interpretations of candidate pathogen identity, pathogenesis, diagnostics, microbiome regulation, and management therefore still depend substantially on evidence from other *Cymbidium* species, other Orchidaceae, greenhouse ornamentals, and general plant-pathology systems. Second, microbial disease problems in protected *C. faberi* production can be provisionally approached within an environmentally and operationally structured disease-complex framework, rather than regarded as a confirmed system of stable multi-pathogen synergistic pathogenesis. This framework links detection, prevention, intervention, and release decisions to specific production stages while making the underlying extrapolations explicit. Third, conversion of this framework into a validated *C. faberi*-specific management system requires pathogen isolation and pathogenicity confirmation, quantitative microclimate and inoculum thresholds, long-term protected-production trials, microbiome-function validation, multi-omics resolution, and prospective testing of data-driven early-warning models. Until these requirements are met, the proposed IDM structure should be used as a provisional decision-support framework, and the validation agenda should guide its calibration and protected-production implementation.

## Supplementary material

Supplementary Table 1 Major disease-management measures and their current applicability to protected *Cymbidium faberi* production.

Descriptive characterization of the 82 scientific publications included in the evidence synthesis of this critical narrative review, including study host, topic, study type, pathogenicity testing, and production-relevant evaluation.
