# Supplementary material for: Evidence boundaries and integrated disease management of microbial disease risks in protected Cymbidium faberi production: a translational framework and validation agenda
Source: Front Plant Sci. 2026 Oct 9;17:1952084. doi: 10.3389/fpls.2026.1952084 (PMC13693643; doi:10.3389/fpls.2026.1952084)
Supplement: Supplementary file 1 [file SupplementaryFile1.docx]

**Supplementary Table S1. Major disease-management measures and their current applicability to protected *Cymbidium faberi* production.**

| **Management measure** | **Target stage/pathogen** | **Expected advantage** | **Main limitation** | ***Current applicability to C. faberi*** | **Relevant references** | **Study context** |
| --- | --- | --- | --- | --- | --- | --- |
| Healthy mother-plant selection, virus elimination, and clean propagation | Preventing pathogen entry / removing infected material | Reducing inoculum at the source | Long detection cycle, high cost | Translatable after local validation | Takahashi et al., 2019; Nongdam et al., 2023; Eun et al., 2000 | OR+MP |
| Targeted molecular and serological detection (PCR, multiplex PCR, qPCR/RT-qPCR, RT-RPA, and ELISA) | Pathogen monitoring and batch release (including *Cymbidium* multi-pathogen) | Sensitive and specific; qPCR/RT-qPCR can provide quantitative information | Assay-specific validation, threshold calibration, and cost control are required | Translatable after local validation (assay and threshold validation required) | Venbrux et al., 2023; Kim et al., 2024; Eun et al., 2000; Ren et al., 2020; Sun et al., 2024 | CY+OR+MP |
| Environmental regulation (ventilation/irrigation/shading) | High-humidity windows/oomycetes/fungi | Reducing disease occurrence probability | Limited by facilities and climate | Operationally feasible in principle, pending local quantitative calibration | Mahmoud et al., 2023; Daughtrey and Buitenhuis, 2020 | GH |
| Chemical control (protective fungicides) | Leaf spot, gray mold, anthracnose | Rapid suppression of early disease | Resistance, ecological disturbance | Operationally feasible in principle, subject to local registration, pathogen identification, and MoA-rotation requirements | Corkley et al., 2022; Guarnaccia et al., 2021 | GH+MP |
| Biological control/antagonistic microbes | Candidate bacterial and fungal diseases; rhizosphere/endosphere applications | Environmentally friendly, sustainable | Efficacy variability; biosafety and host-range uncertainty; formulation and colonization instability | *C. faberi*-derived endophyte screening and cross-species *in vivo* proof of concept are available; protected *C. faberi* production efficacy, biosafety, formulation stability, and management compatibility remain unvalidated. | Bonaterra et al., 2022; Fontana et al., 2021; Teixidó et al., 2022; Jiang et al., 2023; Wen et al., 2025 | CF+CY+MP |
| Tool disinfection and zoning hygiene | Mechanical transmission/operational hygiene | Low cost, easy to operate | Depends on implementation consistency | Operationally feasible in principle; *C. faberi*-specific effect size remains unquantified | Ocimati et al., 2021; Daughtrey and Buitenhuis, 2020 | GH+MP |
| Disease-resistance breeding | Genetic resistance improvement | Can provide more durable host resistance | Long cycle, complex genetic background | Exploratory | Iiyama et al., 2024; Hasan et al., 2021 | OR+MP |
| HTS/metagenomics | Unknown pathogens/complexes | Discovering novel factors and co-infections | High cost, high analysis threshold | Exploratory (research use) | Massart et al., 2017; Nizamani et al., 2023; Maree et al., 2018 | MP |

**Note:** Study context: CF, direct *Cymbidium faberi* studies; CY, other *Cymbidium* species; OR, other Orchidaceae; GH, non-orchid greenhouse or nursery crops; MP, model plants and other general plant-pathology systems. Current applicability to *C. faberi* is a descriptive assessment of translational readiness based on the cited biological and operational information. It does not indicate validated efficacy in protected *C. faberi* production and should not be interpreted as a formal evidence grade.

**Supplementary Table S2. Descriptive characterization of the 82 scientific publications included in the evidence synthesis of this critical narrative review, including study host, topic, study type, pathogenicity testing, and production-relevant evaluation.**

| **Ref** | **Subject / host** | **Pathogen / topic** | **Study context** | **Study type** | **Pathogenicity testing reported** | **Production-relevant evaluation reported** |
| --- | --- | --- | --- | --- | --- | --- |
| [1] Ali et al., 2019 | general plant pathology review | diagnostic methods review (non-destructive detection) | MP | Review | N.A. | N.A. |
| [2] Aman et al., 2020 | CRISPR/Cas biosensing (general) | nucleic acid detection methods | MP | Review | N.A. | N.A. |
| [3] Arnault et al., 2023 | plant microbiota (model/general) | microbiome dysbiosis (Anna Karenina Principle) | MP | Review | N.A. | N.A. |
| [4] Bag et al., 2024 | orchids (general) | *Phytophthora* / oomycetes | OR | Review | N.A. | N.A. |
| [5] Bailey-Serres and Voesenek, 2008 | plants (general; flooding) | flooding/abiotic stress (disease predisposition) | MP | Review | N.A. | N.A. |
| [6] Balotf et al., 2025 | model plants/general | multi-omics of plant-pathogen interactions | MP | Review | N.A. | N.A. |
| [7] Bhunjun et al., 2021 | fungal plant pathogens (general) | fungal molecular ID + Koch's postulates guidelines | MP | Review | N.A. | N.A. |
| [8] Bika et al., 2021 | greenhouse ornamentals | *Botrytis* gray mold | GH | Review | N.A. | N.A. |
| [9] Bonaterra et al., 2022 | plants (general; BCA review) | bacterial biocontrol agents | MP | Review | N.A. | N.A. |
| [10] Buirs and Punja, 2024 | *Cannabis sativa* (greenhouse) | IDM framework / multiple pathogens | GH | Review | N.A. | N.A. |
| [11] Chang et al., 2026 | commercial orchids, including *Cymbidium* spp. (Taiwan) | *Fusarium* (FOSC) leaf blight/stem/root rot | CY | Primary research | Yes | Yes |
| [12] Charkowski, 2018 | general plant pathology review | bacterial soft rot (Pectobacteriaceae) | MP | Review | N.A. | N.A. |
| [13] Corkley et al., 2022 | plants (general) | fungicide resistance management | MP | Review | N.A. | N.A. |
| [14] Dastogeer et al., 2022 | rice | foliar pathogen / microbiome-pathobiome | MP | Primary research | No | No |
| [15] Daughtrey and Buitenhuis, 2020 | greenhouse ornamentals | IDM/IPM framework | GH | Book chapter | N.A. | N.A. |
| [16] Dodds et al., 2024 | model plants/general | plant immunity / NLR resistance genes | MP | Review | N.A. | N.A. |
| [17] Du et al., 2025 | Five Orchidaceae species, including *C. faberi*, from the Funiu Mountains | Rhizosphere bacterial diversity, environmental associations, community assembly, and functional prediction | CF+OR | Primary research | No | No |
| [18] Dutt et al., 2022 | general plant pathology review | multi-infection / pathogen coexistence | MP | Review | N.A. | N.A. |
| [19] Emmenegger et al., 2023 | *Arabidopsis* (model) | SynComs / disease-suppressive microbiome | MP | Primary research | No | No |
| [20] Eun et al., 2000 | *Oncidium* orchids with mixed CymMV/ORSV infection | CymMV/ORSV viruses | OR | Method/Protocol | N.A. | No |
| [21] Fontana et al., 2021 | plants (general) | endophytic fungi / induced resistance | MP | Review | N.A. | N.A. |
| [22] Ghimire et al., 2024 | recycled irrigation water for horticultural nursery systems | *Phytophthora/Phytopythium* inactivation by UV-C treatment | GH | Primary research | No | Yes |
| [23] Gomez-Gutierrez and Goodwin, 2022 | wheat | LAMP detection of wheat pathogens | MP | Review | N.A. | N.A. |
| [24] González-Domínguez et al., 2023 | general plant pathology review | disease models and forecasting | MP | Review | N.A. | N.A. |
| [25] Guarnaccia et al., 2021 | bedding plants (greenhouse) | fungal leaf spot/anthracnose/gray mold | GH | Review | N.A. | N.A. |
| [26] Gullino et al., 2022 | greenhouse/nursery soils | soil disinfestation / soilborne pathogens | GH | Review | N.A. | N.A. |
| [27] Hasan et al., 2021 | plants (general; breeding) | marker-assisted selection (MAS) | MP | Review | N.A. | N.A. |
| [28] Hasanaliyeva et al., 2022 | fruit and foliar crops | disease detection and forecasting | MP | Review | N.A. | N.A. |
| [29] Hinsley et al., 2018 | orchids (general; trade) | orchid trade / conservation | OR | Review | N.A. | N.A. |
| [30] Hönig et al., 2023 | plants (general) | chemical priming of defense | MP | Review | N.A. | N.A. |
| [31] Huang et al., 2020 | *Cymbidium* | *Fusarium oxysporum* pseudobulb rot | CY | Primary research | Yes | No |
| [32] Huang et al., 2022 | Oriental *Cymbidium*, including *C. ensifolium*, *C. sinense*, and *C. goeringii* | *F. proliferatum* fleck spot; pathogenicity, temperature response, cultivar susceptibility, and shoot-age susceptibility | CY | Primary research | Yes | Yes |
| [33] Iiyama et al., 2024 | *Phalaenopsis* | orchid breeding / disease resistance | OR | Review | N.A. | N.A. |
| [34] Ilyas et al., 2022 | plants (general; tobamovirus) | tobamovirus latent infection | MP | Review | N.A. | N.A. |
| [35] Jiang et al., 2023 | *C. faberi* 'Hongxiangfei' (endophyte source); *C. goeringii* 'Green Cloud' (*in vivo* test) | Root endophytic fungi; *in vitro* antagonism against fungal pathogens; cross-species *in vivo* suppression test | CF+CY | Primary research | No | No |
| [36] Kanapiya et al., 2024 | general plant pathology review | plant viral diagnostics | MP | Review | N.A. | N.A. |
| [37] Kim et al., 2024 | *C. kanran* | multiplex PCR detection of *C. gloeosporioides*, *Pestalotiopsis* sp., *F. oxysporum*, and *F. proliferatum* in *C. kanran* | CY | Method/Protocol | N.A. | Yes |
| [38] Lamichhane and Venturi, 2015 | general plant pathology review | pathogen synergism / disease complexes | MP | Review | N.A. | N.A. |
| [39] Lee et al., 2024 | *Phalaenopsis* | orchid viruses (Nerine latent virus; Nanopore/CRISPR detection) | OR | Method/Protocol | N.A. | Yes |
| [40] Leveau, 2024 | general plant pathology review | disease triangle / microbiota integration | MP | Review | N.A. | N.A. |
| [41] Liu et al., 2009 | *C. faberi* from Luanchuan County, Henan, China | Anthracnose-associated fungus; pathogen isolation and identification; *in vitro* inhibition by nine plant extracts | CF | Primary research | No | No |
| [42] Lu et al., 2022 | *Colletotrichum* (model fungal pathogen) | *Colletotrichum* proteinaceous effectors | MP | Review | N.A. | N.A. |
| [43] Luo et al., 2019 | Wild *C. faberi* roots from three Qin-Ba Mountain populations | Culturable root-endophytic fungal diversity and geographic differentiation | CF | Primary research | No | No |
| [44] Lv et al., 2023a | *C. goeringii* and *C. faberi* after introduction/domestication in an orchid garden | Rhizosphere and non-rhizosphere bacterial and fungal community diversity | CF+CY | Primary research | No | Yes |
| [45] Lv et al., 2023b | plants (general; microbiome) | pathogenesis / microbiome | MP | Review | N.A. | N.A. |
| [46] Mahmoud et al., 2023 | *Cannabis sativa* (greenhouse) | *Botrytis cinerea* bud rot | GH | Primary research | Yes | Yes |
| [47] Maree et al., 2018 | general plant pathology review | HTS plant virus diagnostics | MP | Review | N.A. | N.A. |
| [48] Massart et al., 2017 | general plant pathology review | HTS / virus biosecurity framework | MP | Review | N.A. | N.A. |
| [49] Moon et al., 2017 | *Cymbidium* orchids (Korea) | *Burkholderia gladioli* | CY | Primary research | Yes | No |
| [50] Muñoz et al., 2019 | cut flower roses (commercial greenhouse) | *Botrytis cinerea* gray mold | GH | Primary research | No | Yes |
| [51] Murmu et al., 2024 | plants (general; omics) | AI-assisted omics in plant defense | MP | Review | N.A. | N.A. |
| [52] Nizamani et al., 2023 | general plant pathology review | HTS in disease management | MP | Review | N.A. | N.A. |
| [53] Nongdam et al., 2023 | orchids (general) | orchid micropropagation | OR | Review | N.A. | N.A. |
| [54] Ocimati et al., 2021 | banana | *Xanthomonas* wilt / tool sterilization | MP | Primary research | No | Yes |
| [55] Panno et al., 2020 | plants (general; virology) | LAMP in plant virology | MP | Review | N.A. | N.A. |
| [56] Park et al., 2020 | *Cymbidium* orchids (Korea) | *Colletotrichum cymbidiicola* anthracnose | CY | Primary research | Yes | No |
| [57] Possamai and Wiedemann-Merdinoglu, 2022 | grapevine | *Plasmopara viticola* / *Erysiphe necator* (QTL/MAS phenotyping) | MP | Review | N.A. | N.A. |
| [58] Pujasatria et al., 2024 | orchids (general) | OMF / orchid mycorrhiza ISR | OR | Primary research | No | No |
| [59] Redekar et al., 2020 | horticultural nursery (recycled irrigation water) | oomycetes in recycled irrigation water | GH | Primary research | No | Yes |
| [60] Ren et al., 2020 | Symptomatic Chinese *Cymbidium* samples from Guangdong production areas; host species not resolved to *C. faberi* | CymMV and ORSV detection, single and mixed infection, and cp-gene phylogeny | CY | Primary research | No | Yes |
| [61] Romero et al., 2022 | plants (global disease ecology) | fungal disease outbreaks (humidity/temperature) | MP | Review | N.A. | N.A. |
| [62] Sangiorgio et al., 2022 | plants (general) | pesticide effects on phytobiota | MP | Review | N.A. | N.A. |
| [63] Sen et al., 2006 | *Cymbidium* spp. | pseudobulb soft rot complex (*Erwinia*/*Fusarium*/*Mucor*) | CY | First report / Case report | Yes | No |
| [64] Shafay et al., 2025 | general plant pathology review | plant disease detection | MP | Review | N.A. | N.A. |
| [65] Soubeyrand et al., 2024 | general plant pathology review | plant health surveillance | MP | Review | N.A. | N.A. |
| [66] Spök et al., 2022 | genome-edited plants (policy) | genome editing / social acceptability | MP | Review | N.A. | N.A. |
| [67] Spooren et al., 2024 | plants (general; soil microbiome) | disease-suppressive soil microbiomes | MP | Review | N.A. | N.A. |
| [68] Sun et al., 2024 | *Dendrobium, Oncidium, Zygopetalum,* and *Phalaenopsis* orchids | CymMV/ORSV viruses (duplex RT-RPA) | OR | Method/Protocol | N.A. | Yes |
| [69] Takahashi et al., 2019 | plants (general; virology) | virus latency | MP | Review | N.A. | N.A. |
| [70] Teixidó et al., 2022 | biocontrol agents (general) | BCA production/formulation/shelf life | MP | Review | N.A. | N.A. |
| [71] Trivedi et al., 2020 | plants (general; microbiome) | plant-microbiome interactions | MP | Review | N.A. | N.A. |
| [72] Venbrux et al., 2023 | general plant pathology review | plant pathogen detection methods | MP | Review | N.A. | N.A. |
| [73] Venu et al., 2025 | plants (general; virology) | dsRNA-mediated RNAi / viral resistance | MP | Review | N.A. | N.A. |
| [74] Villamor et al., 2019 | general plant pathology review | HTS plant virus detection/discovery | MP | Review | N.A. | N.A. |
| [75] Walsh et al., 2024 | plants (general; phenomics) | imaging sensors + AI stress detection | MP | Review | N.A. | N.A. |
| [76] Wang et al., 2015 | Wild and greenhouse-grown *C. faberi* roots | Culturable root-endophytic bacterial diversity and growth-environment comparison | CF | Primary research | No | Yes |
| [77] Wen et al., 2025 | Wild *C. goeringii* and *C. faberi* rhizosphere soil and roots | Rhizosphere fungal communities; root endophytes and growth-promoting traits | CF+CY | Primary research | No | No |
| [78] Xue et al., 2020 | orchids (4 spp., incl. *Phalaenopsis*) | NLR resistance genes | OR | Primary research | No | No |
| [79] Yang et al., 2021 | *Cymbidium mannii* (and *Epidendrum radicans*) | OMF / orchid mycorrhiza (germination) | CY | Primary research | No | No |
| [80] Yu et al., 2015 | *C. goeringii* and *C. faberi* roots | Culturable root-associated and endophytic fungi, including *Tulasnella*, *Umbelopsis*, *Trichoderma*, and *Fusarium* | CF+CY | Primary research | No | No |
| [81] Zhang et al., 2022 | orchids (general; industry) | orchid industry / research review | OR | Review | N.A. | N.A. |
| [82] Zhou et al., 2021 | orchid (unspecified; Canada) | *Dickeya fangzhongdai* soft rot | OR | First report / Case report | Yes | No |

**Note:** Study context: CF, direct *Cymbidium faberi* studies; CY, other *Cymbidium* species; OR, other Orchidaceae; GH, non-orchid greenhouse, nursery, or protected-production systems; and MP, model plants and other general plant-pathology systems. Classification was based on the host material or production system actually evaluated. Mixed-host orchid studies were classified as CY only when *Cymbidium*-specific results were reported separately; otherwise, they were classified as OR. Pathogenicity testing reported was recorded as Yes when causal validation was explicitly performed and as No when an empirical study did not report pathogenicity testing, regardless of whether such testing was central to the original study objective. A No classification is descriptive and does not imply a methodological deficiency. N.A. was used for reviews, methods/protocols, policy papers, and other non-empirical publication types for which pathogenicity testing was outside the study design. Production-relevant evaluation reported was recorded as Yes when the focal diagnostic method, disease process, environmental relationship, or management intervention was evaluated under greenhouse, nursery, field-production, or other operationally relevant production conditions. Ecological surveys of wild populations, or field sampling conducted solely for microbial-community characterization, were recorded as No. Collection of isolates or symptomatic material alone was insufficient. These classifications describe the original publications and do not represent formal evidence grades, risk-of-bias assessments, *C. faberi*-specific validation, or established management efficacy. Combined context labels were used when distinct components of a study directly evaluated more than one host or evidence context. CF classification indicates direct use of *C. faberi* material but does not imply pathogen causality, disease-specific validation, or established management efficacy. This table summarizes the 82 scientific publications included in the evidence synthesis and should not be interpreted as the output of a formal systematic-review screening process. Contextual institutional sources used solely for trade statistics or conservation status are not included in this evidence table. FOSC, *Fusarium oxysporum* species complex.
